# Strategies for MOF Functionalization in Pyrotechnics and Propellants: Internal and Interfacial Approaches

**DOI:** 10.3390/molecules31193416

**Published:** 2026-09-25

**Authors:** Aigerim Imash, Gaukhar Smagulova, Vladimir Efremov, Kaster Kamunur, Lyazzat Mussapyrova, Aisulu Batkal

**Affiliations:** 1 Institute of Combustion Problems, 172 Bogenbay Batyr Str., Almaty 050012, Kazakhstan; iimash.aigerim@gmail.com (A.I.); vlaefremov@mail.ru (V.E.); kamunur.k@mail.ru (K.K.); lyazzat.mussapyrova@gmail.com (L.M.); abatkalova@mail.ru (A.B.); 2 Faculty of Chemistry and Chemical Technology, Al-Farabi Kazakh National University, 71 al-Farabi Ave., Almaty 050040, Kazakhstan

**Keywords:** metal–organic frameworks (MOFs), energetic materials, pyrotechnic compositions, MOF functionalization, interfacial engineering, structure–property relationships, ignition and combustion, ammonium perchlorate

## Abstract

Metal–organic frameworks (MOFs) provide a modular platform for controlling energetic performance, ignition, combustion, thermal stability, and safety, but the structural origin of these effects is often difficult to distinguish from interfacial contributions in multicomponent pyrotechnic formulations. This review organizes MOF functionalization according to the dominant level of modification: internal-framework functionalization (IF), including linker, metal-node, pore, counterion, and guest engineering, and external/interfacial functionalization (EF), including interfaces with metallic or boron fuels, carbon materials, oxidizers, and polymer matrices. Only systems in which intact MOFs are introduced into the energetic formulation are considered, and representative studies are compared through structure–mechanism–response relationships. The analysis shows that IF primarily governs intrinsic chemical reactivity, redox pathways, energetic content, initiation mode, and sensitivity, whereas EF determines how efficiently this functionality is delivered through heat, mass, and charge transfer and spatial contact. Reported improvements in decomposition, ignition, combustion, and pressure generation are strongly condition-dependent and may involve trade-offs in sensitivity, compatibility, or active-fuel fraction. Functionalization should therefore be selected according to the rate-limiting stage of the target formulation, with matched controls and formulation-level validation required for mechanistic interpretation and practical design.

## 1. Introduction

The development of energetic materials requires simultaneous control of several interrelated characteristics, including energetic performance, ignition, combustion, thermal stability, and sensitivity. Improvement in one property is often accompanied by deterioration in another, reflecting the well-known trade-off among energetic performance, stability, and safety in energetic materials [1,2,3]. In composite propellants, the problem is further complicated by the fact that thermal decomposition of the oxidizer strongly affects ignition and burning rate but represents only one stage of a multistep process; therefore, the performance of the final formulation is governed by a set of coupled physicochemical processes [4,5]. Establishing relationships between material structure and macroscopic energetic properties therefore remains an important objective. Reticular chemistry provides a conceptual basis for constructing extended crystalline frameworks from molecular building units with defined geometries and connectivities [6]. Early studies of isoreticular metal–organic frameworks (MOFs) demonstrated that pore dimensions and chemical functionality could be varied systematically while retaining the underlying network topology [7].

MOFs are particularly promising because the nature of the metal nodes and organic linkers, the coordination environment, pore microenvironment, counterions, and guest components can be tuned independently, while specialized interfaces with external phases can also be constructed. The development of three-dimensional energetic MOFs demonstrated that energy-rich nitrogen-containing fragments and metal centers can be integrated within an ordered architecture [8]. Energetic metal–organic frameworks (EMOFs) have subsequently been considered not only as standalone high-energy or initiating compounds, but also as oxidizer-decomposition catalysts, regulators of ignition and combustion, and functional components of pyrotechnic and rocket-propellant systems [9,10].

Experimental evidence indicates that structural modifications of MOFs can substantially affect energetic properties. For example, incorporation of acetylene groups into an imidazolate linker enabled millisecond hypergolic ignition that differed markedly from the behavior of related frameworks and the corresponding starting components [11]. In composite propellants, the composition of Cu-containing MOFs affected component decomposition, aluminum agglomeration, and combustion behavior [12]. Changes in electronic structure also produced pronounced effects: for the mixed-valence Cu(II)/Cu(III)-CuBTT framework, the high-temperature decomposition peak of ammonium perchlorate (AP) was reported to decrease by 127.2 °C, while heat release increased by 81.6% [13]. Thus, the energetic response of MOFs depends both on the framework structure itself and on its interactions with the surrounding formulation components.

Despite substantial progress in understanding structure-property relationships in EMOFs, establishing an unambiguous link between a specific structural modification and an observed energetic response remains challenging [9,10,14]. A decrease in AP decomposition temperature, a shorter ignition delay, or faster combustion may arise from linker chemistry, the identity and valence state of the metal node, the composition of the pore environment, or interactions of the MOF with the fuel, oxidizer, and binder. Moreover, improvement in one metric may be accompanied by deterioration in another, for example, through increased mechanical or electrostatic sensitivity. It is therefore important to consider not only the magnitude of an effect but also the structural level at which it originates.

Existing reviews of EMOFs predominantly classify materials according to synthesis and structural features, energetic characteristics, and areas of application [10,14]. More application-focused reviews have separately addressed the construction and applications of nitrogen-rich EMOFs, EMOF-based burning-rate catalysts for solid propellants, and metal-coordinated hypergolic materials [15,16,17]. However, these studies do not explicitly organize energetic-MOF functionalization according to whether the dominant structural modification occurs within the intact framework or at its interface with another energetic component. In the broader MOF literature, functionalization is commonly discussed in terms of modifications to organic linkers, metal nodes, the internal pore environment, or the external framework surface [18,19,20,21]. Early experimental work demonstrated that different organic functionalities could be introduced selectively into the interior and onto the exterior of the same MOF crystals [22]. This spatially selective modification provides an experimental basis for distinguishing internal functionalization from external surface chemistry. However, for energetic MOFs, these levels of functionalization are rarely integrated into a single framework that connects the location of a structural change with its mechanism of action and the measured energetic response. Accordingly, this review adopts a classification based on the structural level at which the dominant variable is modified.

Internal-framework functionalization (IF) includes modifications of linkers, metal nodes, the pore environment, counterions, and guest components. External and interfacial functionalization (EF) refers to systems in which the response is governed primarily by interactions between the MOF and an external phase, such as a metallic or boron-containing fuel, carbon material, oxidizer, polymer binder, or energetic matrix.

The aim of this review is therefore to establish relationships among the level of structural functionalization, the governing mechanism, and the measured energetic property. Particular attention is given to three questions: which internal-framework modifications affect energetic-material performance, when interfacial interactions become dominant, and which control experiments can distinguish between these mechanisms.

## 2. Internal-Framework Functionalization of Intact MOFs for Pyrotechnics and Propellants

Internal-framework functionalization involves deliberate modification of structural and chemical elements within the MOF itself while retaining the parent framework architecture before incorporation into an energetic system. The principal tunable variables include organic-linker chemistry, the identity and ratio of metal nodes, the composition of the pore microenvironment, counterions, and guest components [18,19]. Modification of these parameters can tune the electronic and coordination structure of the framework, the content of energetically active fragments, redox properties, thermal stability, ignition, and sensitivity of energetic materials [9,10].

Internal functionalization of metal–organic frameworks can therefore be divided into several approaches: modification of the organic ligand, variation in metal-node composition, engineering of the pore space, incorporation of guest components, and coupled modification of several structural elements. These relationships and representative examples are summarized schematically and comparatively (Figure 1; Table 1).

### 2.1. Linker Functionalization: Electronic Tuning, Energetic Contribution, and Sensitivity

The organic ligand in an energetic MOF performs functions beyond simply linking metal nodes. Substituent replacement can simultaneously alter the electron density at the coordination center, the intrinsic fuel value of the framework, molecular packing, and sensitivity to external stimuli. The most informative question is therefore not which ligand provides the highest energy, but which stage of the energetic transformation it predominantly governs: initiation, reaction propagation, or material stability before initiation [11,23,32,33,34,35,36,37,38,39,40].

The most direct effect of linker chemistry is observed during initiation. In isostructural Zn-, Co-, and Cd-ZIFs, replacing a vinyl-substituted imidazolate linker with an acetylene-containing analogue shortened the hypergolic ignition delay from 11–35 ms to 2–5 ms, whereas the free acetylene-containing ligand ignited much more slowly, in approximately 34 ms [11].

A particularly direct post-synthetic linker-functionalization strategy was demonstrated for the acetylene-bearing Zn(AIm)_2_ framework, in which energy-rich 1,2-dicarbadodecaborane-1-thiol (CB–SH) was covalently incorporated through thiol–yne click chemistry. The resulting functionalized framework exhibited a 46.6% increase in energy density and a six-fold decrease in hypergolic ignition delay to 4 ms with red fuming nitric acid, while also showing improved stability [41]. A related electronic-tuning effect was observed in a Cu(I)-MOF: shifting the Cu d-band center from −3.93 to −2.92 eV was accompanied by a decrease in the ignition delay of the [BMIM]N(CN)_2_/H_2_O_2_ system from 120 to 18.4 ms and by the formation of •OH and ^1^O_2_ [23]. These findings suggest that a reactive group is not effective in isolation; rather, the coordination environment modulates charge transfer and thereby influences the rate of the initial chemical response.

A second role of the ligand is to modify the framework’s intrinsic energetic contribution. In the isostructural ANA-Zn(EtIm)_2_/ANA-Zn(PrIm)_2_/ANA-Zn(BuIm)_2_ series, lengthening the alkyl substituent increased the measured gravimetric combustion energy from 24.3 to 28.0 kJ g^−1^ and the volumetric combustion energy from 26.5 to 36.3 kJ cm^−3^ [32]. However, when the framework interacts with an external oxidizer, the relationship is no longer governed by a single variable. Thus, conditions that accelerate subsequent decomposition and combustion do not necessarily facilitate formation of the first reactive center.

This distinction is particularly important in systems in which the MOF itself becomes a precursor to an active phase upon heating. UiO-66-NH_2_ substantially shifts the main AP decomposition peak and increases heat release [33], but forms ZrO_2_ during heating. The NH_2_ functionality may therefore influence not only the initial MOF-AP interaction but also the trajectory of subsequent phase evolution. In such cases, the ligand should be viewed as a means of programming the sequence of transformations rather than as a static catalytic element. This distinction explains why the combustion energy of the framework, the oxidizer decomposition temperature, and actual ignition should not be treated as equivalent performance metrics [32,33,34].

For standalone energetic MOFs, the central issue is the balance between high reactivity and resistance to accidental initiation. Rigid N-rich coordination networks show that these requirements can coexist: IFMC-1 retains an impact sensitivity (IS) > 40 J, friction sensitivity (FS) > 360 N, and electrostatic-discharge sensitivity (ESD) > 44 J at a decomposition temperature (Td) of approximately 392 °C [35], while tetrazole-based K-MOFs and three-dimensional [Cu(DNBT)(ATRZ)_3_]_n_ exhibit similarly low mechanical sensitivity together with high calculated energetic characteristics [36,37]. A related Ag(I) three-dimensional framework assembled from combined furazan and tetrazole motifs also exhibited low impact sensitivity (IS > 40 J) together with high calculated detonation performance [42]. The reported desensitization is likely related not simply to a high nitrogen content but to the incorporation of nitrogen-rich units into a rigid coordination network that restricts structural degrees of freedom and hinders localization of mechanical energy.

Coordination locking, however, does not guarantee a favorable sensitivity profile. K_2_TNAP, with calculated detonation velocity D = 8.215 km s^−1^ and detonation pressure P = 30.7 GPa, has IS = 4 J and FS = 10 N [38], whereas an azide-tetrazole Zn-MOF with a high nitrogen content shows more moderate calculated values of D = 5.96 km s^−1^ and P = 9.56 GPa [39]. For Pb(bta)·2H_2_O, the high density of 3.250 g cm^−3^ contributes to calculated values of D = 8.963 km s^−1^ and P = 43.47 GPa while maintaining IS > 40 J [40]; however, this performance is strongly associated with the heavy Pb center and is therefore not readily transferable to lower-toxicity systems. These examples indicate that N-rich chemistry, packing density, and sensitivity are independent design variables rather than parameters that vary along a single scale of energetic performance.

Thus, linker functionalization acts through coupled electronic and structural mechanisms rather than simply by increasing the number of energetic groups. Electron-donating or electron-withdrawing substituents modify ligand–metal orbital coupling and the local electron density at the coordinated metal center, thereby shifting charge-transfer energetics and changing the adsorption and activation of oxidizing species. In the Cu(I)-MOF series, for example, replacing the more electron-withdrawing nitro environment with sulfur-containing coordination shifted the Cu d-band center toward the Fermi level, which strengthened reactant adsorption and facilitated H_2_O_2_ activation; radical analysis identified •OH, •O_2_^−^, and ^1^O_2_ as reactive intermediates [23]. Linker chemistry also influences lattice packing and mechanical sensitivity. Several N-rich energetic MOFs combine rigid coordination architectures with low measured impact and friction sensitivity [35,36,37]. However, this relationship is not universal, as other energetic frameworks with similarly dense or highly coordinated structures can remain substantially more sensitive [38,39,40]. These comparisons indicate that sensitivity depends on the combined effects of linker chemistry, coordination geometry, packing, and intermolecular interactions rather than on energetic-group content alone. Linker functionalization therefore controls initiation, energy release, and sensitivity through the combined effects of electronic redistribution, reactive-intermediate formation, and lattice organization.

### 2.2. Metal-Node Functionalization: Redox Chemistry and Metal Cooperation

Metal-node functionalization primarily changes the redox pathway by altering accessible oxidation states, electron-transfer kinetics, adsorption of decomposition intermediates, and the density of catalytically active centers while the ligand environment is retained as closely as possible. In AP-containing systems, the initial decomposition involves electron-transfer processes that generate HClO_4_- and NH_3_-derived intermediates; accumulation of adsorbed NH_3_ can retard further decomposition. Redox-active metal nodes can lower this kinetic barrier by facilitating electron transfer and by promoting adsorption and subsequent conversion of these intermediates. For Fe/Co-containing MOFs, for example, Fe and Co redox couples accelerate charge transfer, while oxygen-deficient metal–oxygen environments provide adsorption sites for NH_3_ and related gaseous intermediates. TG–IR analysis of Fe–Co-MOF/AP confirmed enhanced charge transfer and NH_3_ adsorption, together with altered conversion of N_2_O/NO_x_ products [24]. Consequently, changing the node identity can modify not only the apparent activation energy and decomposition temperature but also the reaction pathway itself. This effect is most reliably evaluated within matched series in which the ligand, loading, and test conditions are held constant.

In the [M(EIM)_2_(BTB)_2_]_n_ series at a 5 wt.% loading, Co-, Cu-, and Cd-MOFs lowered the high-temperature decomposition (HTD) peak of AP from 393.6 °C to 330.4, 342.1, and 387.7 °C, respectively, and reduced the apparent activation energy (E_a_) from 196.7 kJ mol^−1^ to 130.4, 152.0, and 187.8 kJ mol^−1^ [43]. In a different dicyanamide (DCA) series at 10 wt.%, Co-, Cu-, and Pb-MOFs produced HTD values of 318.3, 306.5, and 340.3 °C, respectively, compared with 379.2 °C for AP [25]. An independent matched-metal comparison based on the energetic H_2_AzTO ligand provides the same type of evidence: Co-, Cd-, Ni-, and Cu-containing frameworks were synthesized within one material family, and all combined energetic character with promotion of AP decomposition. Within this series, Cu-AzTO showed the most pronounced catalytic response, with an AP decomposition peak of 298.9 °C reported at a 10 wt.% loading [44]. A later H_2_BTA-based Co/Mn/Cu series likewise showed substantial changes in AP decomposition, including an HTD decrease of up to 67 °C, an increase in total heat release from 535.9 to 2435 J g^−1^, and a decrease in apparent activation energy from 91.9 to 72.1 kJ mol^−1^ [45]. This metal-node dependence also extends beyond AP. In the isostructural [M(DCA)_2_(1-VIM)_2_]_n_ series (M = Mn or Zn), tested with potassium perchlorate at a 5 wt.% loading, the Mn framework decreased the HTD from 606.4 to 424.1 °C and increased heat release from 349.9 to 1071.0 J g^−1^, whereas the Zn analogue gave an HTD of 465.2 °C and a heat release of 984.1 J g^−1^ [46]. These datasets should not be combined quantitatively because of differences in linker chemistry, loading, and the baseline behavior of AP; however, within each series, the electronic nature of the metal clearly alters the decomposition pathway. Likely controlling factors include the accessibility of redox transitions and the ability of metal-containing centers to support electron transfer among HClO_4_, NH_3_, and oxygen-containing intermediates [4,5].

Even within a single series, different metrics may identify different ‘optimal’ nodes. For Co/Cu/Pb-DCA, the lowest HTD was observed for the Cu-MOF, the highest heat release for the Co-MOF, and the lowest apparent E_a_ was calculated for the Pb-MOF [25]. At the same time, the increase in density from approximately 2.03 g cm^−3^ for the Co/Cu analogues to 3.373 g cm^−3^ for the Pb analogue was accompanied by a decrease in molar heat of combustion to 996.7 kJ mol^−1^. Density, the differential scanning calorimetry (DSC) peak position, heat release, and formal E_a_ therefore probe different parts of the reaction network; none of them alone provides a complete ranking of catalytic effectiveness.

The transition to combustion of a complete formulation shifts the emphasis even further. In the Co/Cu/Cd series, all three MOF fuels increased the burning rate to 8.1–13.4 mm s^−1^ compared with 7.1–9.4 mm s^−1^ for the Al-containing control, with the Cu system showing particularly stable rapid combustion [43]. Once the reaction begins, the contribution of the metal node is superimposed on the intrinsic heat release of the N-rich framework, gas generation, and interfacial heat transfer. Consequently, the node that produces the largest HTD shift does not necessarily provide the best macroscopic combustion behavior.

A particularly convincing mixed-node example is provided by a matched series of Fe-, Co-, and Fe–Co-MOFs, which produced the lowest HTD of 298.6 °C compared with both monometallic analogues; heat release simultaneously increased to 1213 J g^−1^, while E_a_ decreased to 151.6 kJ mol^−1^ [24]. In the formulated propellant, Fe–Co-MOF increased the burning rate by 43.8%, compared with 32.1% and 29.5% for the Fe and Co analogues, respectively, and shortened the ignition delay by 71 ms. Agreement among several independent responses makes this result more persuasive than a DSC comparison alone and is consistent with cooperation between Fe/Co redox pathways together with improved spatial accessibility of active centers.

The mere presence of two metals, however, does not demonstrate internal-framework synergy. In a mechanochemically prepared Co–Fe system based on sulfosalicylic acid, strong decreases in AP and AP/Al decomposition temperatures and E_a_ were accompanied by the presence of CoFe_2_O_4_, Co_3_O_4_, and α-Fe_2_O_3_ in the starting material itself [47]. The observed effect is therefore more appropriately assigned to a MOF-dominated multiphase system. This distinction is essential for node engineering: if a separate active phase is already present before the reaction, its contribution cannot be attributed solely to framework-node chemistry. Two comparatively well-defined modes of node engineering—variation of a single metal center and formation of a mixed Fe–Co node—are shown directly (Figure 2) [24,43].

Overall, the metal node should be regarded not as an isolated ‘catalyst’ but as a regulator of the energetic transformation pathway. The most convincing designs are those in which improved redox kinetics are retained when moving from model thermal analysis to actual combustion.

### 2.3. Functionalization of the Internal Microenvironment: Counterions, Guest Components, and Hydration

Functionalization of the internal microenvironment modifies MOF properties without forming a separate external phase. Counterions, guest molecules, localized metals, and coordinated water can alter packing, free volume, internal charge balance, and charge transport. Their effects appear across different physical regimes—from detonation energetics to AP catalysis and controlled initiation. Thus, meaningful comparison is most reliable within matched material series.

A distinct host–guest regime arises when a conventional energetic molecule itself is confined within the MOF pore system. RDX has been encapsulated in nanoporous Zr- and Cr-based MOFs, where the framework retained its morphology after guest incorporation and the encapsulated explosive showed lower impact and friction sensitivity than unconfined RDX and corresponding physical mixtures. The effect was attributed to nanoscale confinement, reduction in internal hotspots, and mechanical shielding by the rigid porous framework [48]. This example extends internal-microenvironment functionalization beyond counterions and molecular initiators and demonstrates that the pore space can directly regulate the safety response of a conventional nitramine without chemical modification of the explosive molecule.

Counterion exchange in cationic Cu–ATRZ frameworks provides one of the clearest examples. Replacing nitrate with dinitramide increased the density from 1.64 to 1.78 g cm^−3^ and the calculated heat of detonation (Q_det_) from 4562 to 7176 kJ kg^−1^, but simultaneously decreased IS from 16 to 9 J, FS from 112 to 73 N, and ESD from 24.75 to 1.9 J [26]. A broader series of azolate and azolate-N-oxide counterions gave calculated Q_det_ values of 4923–7375 kJ kg^−1^ together with substantially different sensitivity thresholds [49]. The energetic guest therefore acts not only through its own enthalpy; packing density, hydrogen bonding, and cavity geometry change simultaneously. Rational host–guest design should therefore restrict the mobility of the energetic anion without suppressing the required reactivity.

The internal microenvironment can also program the catalytic pathway. For Pb-BTC, incorporation of Co-, Ni-, Zn-, and Mn-containing components at the same loading further modified the AP HTD relative to the parent framework: the lowest temperature was observed for the Co-containing variant, whereas the lowest E_a_ was obtained for the Mn system [50]. The mismatch between these criteria confirms that the DSC peak position and a formal kinetic barrier describe different stages. Pore functionalization may therefore affect not only the initial active centers but also the spatially controlled formation of metal-oxide phases during heating.

A different class of effects involves the creation of a new initiation channel rather than an increase in bulk energy. In Ag–ATRZ frameworks, internal BrO_3_^−^ and ClO_4_^−^ species enabled a response to an 808 nm laser with delays of 1.14 and 0.15 ms, respectively, whereas the parent framework and free ATRZ did not initiate [51]. Laser-responsive behavior is not restricted to oxidizing guest anions. Two solvent-free Pb(II) hydroxytetrazolate frameworks with the same three-dimensional topology were directly initiated by laser irradiation, with the response captured by high-speed imaging and reported initiation thresholds below 7 mJ [52]. The rapid response, however, was accompanied by very high mechanical sensitivity. By contrast, encapsulation of an ionic liquid in Ni-MOF acted through electrical conductivity: at the same loading of 0.5 wt.%, the ignition delay of the electrically controlled propellant decreased from 1.158 s for Ni-MOF to 0.722 s for IL@Ni-MOF, and at 1.5 wt.% it reached 0.336 s [27]. These systems illustrate a broader principle: an internal guest can convert a specific external signal—optical or electrical—into localized initiation without universally enhancing all energetic characteristics.

Hydration and the ionic environment provide another control variable. pH-controlled displacement of water from Cu-, Co-, and Zn-containing NTO/ATRZ frameworks changed the calculated Q_det_ values and detonation velocities while also altering the nature of the first thermal events; for the Co system, the early signal was associated with removal of residual water and is therefore not directly comparable with the exothermic decomposition of the Cu and Zn analogues [53]. Similarly, in NH_3_OH^+^-containing Cu- and Mn-bis(tetrazolyl)methane structures, nearly identical experimental heats of combustion—11.447 and 11.510 MJ kg^−1^—were accompanied by markedly different sensitivities: the Mn analogue retained IS > 40 J, FS > 360 N, and ESD > 20 J, whereas the Cu analogue was substantially more sensitive [30]. These results show that the sensitivity profile of a host–guest material is governed not by the guest energy alone but by how the guest is incorporated within the specific coordination and hydrogen-bonding environment.

A complementary limiting case is provided by the solvent-free three-dimensional [Pb(HBTI)]_n_ framework, which contains no coordinated or guest solvent molecules and remains thermally stable up to approximately 325 °C while retaining favorable sensitivity and calculated energetic characteristics. Its ability to promote AP decomposition further shows that the internal microenvironment must also be considered when the relevant structural feature is the absence, rather than the incorporation, of guest species [54].

Pore/guest functionalization should therefore be viewed as control of the local chemical environment rather than simply as a means of increasing the energetic content of the pores. Counterions regulate energetic output and sensitivity, localized metals influence the catalytic pathway, ionic liquids and oxidizing guests define the mode of initiation, and hydration affects packing and the fraction of active mass. The strongest conclusions arise when a functionalized framework is compared with its own nonfunctionalized analogue under the same test conditions.

### 2.4. Coupled Internal-Framework Functionalization: Interdependent Design

Coupled internal-framework functionalization occurs when several internal variables change simultaneously, and their individual contributions cannot be reliably separated without reconstructing the material. In such systems, metal identity, energetic ligand, coordination geometry, hydration, and packing form a single reaction environment. It is therefore particularly important to distinguish universal ‘synergy’ from an advantage that is demonstrated only for a specific measured response [28,29].

Earlier Cu/tetrazole–triazole frameworks illustrate why such coupling is difficult to deconvolute: changes in Cu oxidation state, coordination geometry, framework dimensionality, and guest-water content occur together and are accompanied by changes in thermal and calculated energetic characteristics [55]. A related example is provided by Pb- and Ba-based frameworks constructed from a ligand combining furazan and tetrazole motifs, where metal identity, hydration, coordination environment, and packing vary simultaneously [52].

A more mechanistically resolved example of coupled ligand–metal control is provided by [Co(DCA)_2_(Imat)_2_]_n_ and [Cu(DCA)_2_(Imat)_2_]_n_. At a 3 wt.% loading, the Co framework decreased the AP decomposition peak from 439.3 to 325.5 °C, increased heat release from 537 to 1301 J g^−1^, and narrowed the exothermic peak to only 8 °C. Combined spectroscopic and electronic-structure analyses associated this concentrated heat release with accelerated proton-coupled electron transfer involving strong Co-3d interaction with the reductive DCA^−^ ligand [56].

A complementary two-ligand/two-metal series based on AZTO and AOTO further illustrates this coupling. Across the corresponding Ag- and Cu-containing energetic MOFs, the Cu frameworks produced the stronger AP-promoting response; in particular, AOTO-Cu·4H_2_O decreased the AP HTD by 102.7 °C and reduced the apparent activation energy by 125 kJ mol^−1^ [57]. Because ligand chemistry and metal identity vary within this family, the observed ranking is better interpreted as a coupled coordination-environment effect than as an isolated metal trend.

In [MnCo(EIM)_2_(DCA)_2_]_n_, the mixed Mn/Co composition did not maximize gravimetric combustion energy: 24.34 kJ g^−1^ lay between the corresponding Mn- and Co-based values of 25.81 and 22.31 kJ g^−1^, respectively. Its higher density, however, produced the maximum volumetric combustion energy of 35.93 kJ cm^−3^ while retaining IS > 40 J and FS > 360 N [28]. In the AP/RDX system, the same framework produced a particularly pronounced kinetic effect for RDX, decreasing the apparent activation energy from 153.0 to 86.3 kJ mol^−1^. The applicability of coupled internal design to a broader range of nitramines is further demonstrated by a heteronuclear Cu(II)/Fe(III) energetic MOF based on tetrazole-1-acetic acid. At a 5 wt.% loading, this framework decreased the decomposition-peak temperatures of RDX, HMX, CL-20, and AP by 1.9, 5.8, 5.7, and 71.4 °C, respectively, while the corresponding apparent activation energies decreased by 38.5, 38.3, 63.9, and 27.0 kJ mol^−1^ [31]. The relatively small peak shifts observed for the nitramines despite substantial changes in apparent kinetic barriers further demonstrate that decomposition temperature alone does not fully describe the influence of an internally engineered MOF on energetic-material reactivity.

A different type of coupling is observed in Na-, K-, Cs-, and Rb-BNTA frameworks [29]. Despite similar decomposition-onset temperatures of approximately 283–287 °C, mechanical sensitivity varies substantially: the Na and K analogues retain IS > 40 J and FS > 360 N, whereas the Cs and Rb systems are considerably more sensitive. K-BNTA, with a density of 1.941 g cm^−3^, combines low sensitivity with calculated D = 8844 m s^−1^ and P = 26.88 GPa. Because changing the alkali metal simultaneously alters coordination number, water content, hydrogen bonding, and packing, an isolated ‘cation effect’ cannot be extracted. The observed sensitivity behavior is more likely governed by the collective mechanics of the lattice; namely, how coordination and intermolecular interactions distribute a local perturbation. These examples show that coupled internal design is valuable not because it maximizes a single parameter, but because it can coordinate several functions within one architecture.

Synergy should therefore be considered established only for a response that genuinely exceeds structurally comparable controls; the same system may show a pronounced kinetic advantage while providing only modest gains in energy or sensitivity.

Overall, internal-framework functionalization determines the chemical preparedness of a MOF for reaction. The ligand regulates electronic structure and energetic content, the metal node directs the redox pathway, the internal microenvironment governs local charge, transport, and the mode of initiation, and coupled design links these levels. However, properties generated within the framework must still be expressed in a multicomponent formulation. This shifts the focus toward external and interfacial functionalization, where the spatial organization of contact between the MOF and the fuel, oxidizer, conductive phase, or matrix becomes decisive.

## 3. External and Interfacial Functionalization of Intact MOFs for Pyrotechnics and Propellants

External and interfacial functionalization begins when the primary object of control is no longer the chemistry of an isolated framework but the boundary between the MOF and another phase. Heat, charge, oxidizer, and decomposition products cross this boundary, where disruption of passivating shells, particle agglomeration, accessibility of ignition sites, and compatibility with the matrix are also determined.

External/interfacial functionalization is therefore more usefully classified by interface function than by MOF composition: contact with Al or B governs access to the metallic fuel; carbon supports control transport and dispersion; direct coupling with AP defines the reaction-zone structure; and polymer matrices determine how much of the MOF activity is retained after formation of the final composition. This logic is summarized schematically and comparatively (Figure 3; Table 2).

### 3.1. MOF Interfaces with Metallic and Boron-Containing Fuel Particles

For Al and B, the key interfacial limitation is imposed by the passivating oxide. Al_2_O_3_ increases the diffusion distance to the metallic core, whereas viscous B_2_O_3_ can re-cover the active surface after oxidation has already begun [71]. A MOF shell is beneficial only when it modifies this transport regime—for example, by creating reaction channels, localizing early heat release near the fuel surface, or generating active products directly at the interface [58,59,67,72,73,74,75,76].

For nano-Al, the strongest evidence comes from systems in which a deliberately engineered contact was compared with a physical mixture. In polydopamine (PDA)-assisted n-Al@Cu-EMOF, the framework was grown directly on the Al surface, and the total heat release reached 4142 J g^−1^ [58]. Here, PDA does more than simply bind the components: it defines where Cu-containing products form after MOF decomposition. Early energy release from the shell and the subsequent thermite-like reaction therefore occur within essentially the same interfacial zone, unlike in a random mixture of the same components.

An even more direct approach is represented by n-Al@MIL-53(Al), in which part of the native Al_2_O_3_ layer is used to form the surface MOF. The minimum CO_2_-laser ignition delay decreased from 20 to 4 ms, while the maximum pressure-rise rate in the optimized series increased by nearly one order of magnitude [67]. Physical mixtures of n-Al with either the ligand or the MOF did not reproduce this response. The advantage is consistent with a chemically continuous pathway through the passivation layer: as the shell decomposes, gas and voids are generated exactly where they can shorten the oxidizer transport path to Al [67]. At the same time, the heat of combustion decreases from 22.32 to 17.06 kJ g^−1^, illustrating a trade-off in which faster access to the metal is achieved at the expense of a lower active-metal mass fraction (Figure 4a,b).

Core–shell Al@Ag-MOF provides an independent hypergolic validation of the same interfacial design principle. Direct growth of a hypergolic Ag-MOF shell on Al produced shorter ignition delays and more efficient Al combustion than a simple physical mixture of Al and Ag-MOF, which was attributed to more effective hotspot generation and interfacial heat transfer. The resulting solid hypergolic system also exhibited reported specific impulses of 230–270 s over a broad oxidizer-to-fuel ratio range [77].

A similar principle is observed for poly(vinylpyrrolidone)-assisted n-Al@Zn-MOF and n-Al@Co-MOF: at 3 MPa O_2_, the pressure-rise rate increased by approximately 35–38%, although the heat of combustion was slightly lower than that of pristine Al [76]. These observations are consistent with a redistribution of energy release in time, accelerating its release rather than necessarily increasing the overall gravimetric energy content (Figure 4c,d). A related trend is seen for (3-aminopropyl)triethoxysilane (APTES)-mediated Cu-ATRZ@Al: at the same 5 wt.% loading, the chemically assembled system lowered the AP HTD to 319.1 °C compared with 343.8 °C for the physical mixture and simultaneously produced higher heat release [72]. Here, discrete anchoring of Al may preserve more exposed fuel surface than a continuous coating while keeping Cu redox sites in close proximity.

For B, the interfacial challenge is different: after heating begins, B_2_O_3_ must not only be disrupted but also prevented from re-forming a continuous viscous barrier. In B@PDA@R-MOF(MA), an azide-containing shell increased combustion completeness from 60.7 to 80.4% [59]. Early gas generation and local pressure pulses may disrupt the B_2_O_3_ layer, while the resulting Zn/Co oxides may support subsequent O_2_ transport [59]. The energetic ligand and metal node may therefore contribute at different temporal stages of the same interfacial process.

A systematic nB@MOF series with Zn, Co, and Mo nodes provides further evidence for this two-stage interfacial mechanism. Combustion of the organic ligands promoted micro-explosion and disruption of the boron oxide barrier, whereas metal oxides generated in situ on the B surface facilitated subsequent oxidation. Relative to pristine nB, the maximum pressurization rate increased by 31.1-fold, the initial oxidation temperature decreased by as much as 185.9 °C, and the combustion heat of all investigated nB@MOF systems increased by more than 1.2-fold [66].

Physically assembled nB/EMOF demonstrates that the optimal MOF fraction depends on the stage being measured [75]. In air, the strongest DSC response occurred near 10 wt.% EMOF; in O_2_, the maximum calorimetric heat release was obtained at 25 wt.%; and the fastest flame propagation occurred at 30 wt.%. These maxima are not contradictory: the rate-limiting step changes among regimes—from O_2_ access to the fraction of active B or the intensity of gas generation. MOF concentration therefore cannot be optimized independently of the target metric.

Not all interfaces are intended to accelerate reaction. For flake-μAl, a porphyrinic Al-TCPP shell primarily suppressed chloride-induced corrosion and, after prolonged exposure to 3.5 wt.% NaCl, preserved the phase composition and most of the combustion heat [74]. In a different mode, n-Al/ZIF-67 was used as a microwave-responsive interface: maximum microwave absorption and minimum ignition delay of a double-base propellant occurred for different coating variants [73]. Thus, even within a single class of Al/MOF systems, the function of the interface can differ substantially—reaction acceleration, stabilization during storage, or conversion of an external field into local heating.

Overall, fuel-particle functionalization is most effective when the MOF addresses a specific transport limitation at the fuel surface. For nano-Al, this usually involves shortening transport through Al_2_O_3_ and accelerating pressurization; for B, the reported architectures show a progression from single MOF shells to node-tunable nB@MOF interfaces and ternary B/polymer/MOF systems. In the latter case, the design objective evolves from simple disruption of B_2_O_3_ toward coordinated control of ligand-driven micro-explosion, in situ catalytic-oxide formation, interfacial mass transfer, and fuel accessibility [59,66,75,78]. For micro-Al, the relevant function may instead be preservation of the fuel surface during aging. The key parameter is therefore not the absolute HTD value, but the extent to which the engineered interface modifies the target response while retaining a sufficient fraction of active fuel.

### 3.2. Carbon Supports and Conductive MOF Heterointerfaces

The carbon phase in a MOF composite acts as more than a support. Graphene oxide (GO), reduced graphene oxide (rGO), carbon nanotubes (CNTs), and carbon fibers (CFs) can alter framework nucleation, suppress agglomeration, create pathways for heat and charge transport, and thereby affect different stages of the reaction. Their role is therefore determined not simply by the identity of the carbon material but by the architecture of the MOF/carbon interface and by which process becomes less rate-limiting [60,61,68,79].

One limiting case is stabilization of the framework itself. In a Zn-5-aminotetrazolate MOF, incorporated GO preserved a metastable polymorph after 24 h at 120 °C, whereas physical mixing with GO did not produce the same effect [60]. The calculated Q_det_ changed only moderately. The contribution of GO in this system therefore appears to be predominantly structural rather than energetic: the carbon surface may redirect nucleation and stabilize a local coordination configuration. For an energetic MOF, this example shows that interfacial functionalization can be valuable even without a large increase in energetic metrics if it stabilizes the required phase.

A second carbon-interface regime arises when the support controls EMOF nucleation and electrical initiation rather than AP decomposition. Stepwise coordination of Cu-AFTO on graphene oxide or amino-functionalized graphene oxide reduced the characteristic crystal size from hundreds of micrometers to the nanoscale, while the conductive carbon support simultaneously lowered sensitivity and the electric-ignition threshold [80]. This example directly links interfacial control of crystal growth with a stimulus-specific energetic response.

During AP decomposition, the carbon network primarily acts as a transport architecture. In a Cu-MOF series, CNT- and rGO-containing hybrids produced larger HTD shifts, whereas GO gave the maximum heat release [61]. For the CNT–rGO hybrid, the strongest temperature response occurred without the highest Brunauer–Emmett–Teller surface area, suggesting that network connectivity is more important than surface area alone. One-dimensional CNTs and two-dimensional rGO may jointly create percolation pathways for rapid redistribution of local heat and charge among Cu sites, whereas GO shifts the balance between reaction rate and conversion completeness.

GO@MOF-5 further illustrates this distinction. At a 5 wt.% loading, pristine MOF-5 and GO@MOF-5 produced nearly identical AP HTD values of 322.5 and 321.9 °C, respectively, but incorporation of GO increased ΔH from 815.8 to 1011 J g^−1^ and decreased E_a_ from 139.6 to 84.65 kJ mol^−1^ [68]. Thus, the carbon interface may have little effect on the temperature at which the main reaction is initiated while substantially accelerating subsequent stages. This is an important example of why similar DSC peak temperatures do not imply the absence of an interfacial effect.

With carbon fibers, the transport length scale changes. In ZnCo-ZIF-67/CF, the framework was formed directly on the functionalized fiber surface; at a 5 wt.% composite loading, AP HTD decreased from 424.3 to 337.2 °C, and the composition of nitrogen-containing gaseous products changed substantially [79]. The continuous fiber may provide long-range heat transport and spatially separate MOF crystallites, while Zn/Co centers determine the local redox chemistry. However, without a complete set of separate CF and ZnCo-ZIF controls, the additional contribution of the fiber itself cannot be quantified.

Accordingly, the carbon-interface EF does not define a universal GO–rGO–CNT–CF ranking of effectiveness. The carbon phase may stabilize a metastable framework, create percolation pathways, alter post-initiation kinetics, or distribute active centers over a macroscopic support. The informative variable is therefore not the carbon type alone, but the match between interface topology and the rate-limiting stage of the energetic process.

### 3.3. MOF Interfaces with Oxidizers and Energetic Crystals: Contact, Reaction Zone, and Decomposition Kinetics

Direct coupling of a MOF with AP creates a reaction zone in which electrons, heat, and volatile decomposition products are transferred simultaneously. A decrease in AP decomposition temperature alone does not demonstrate interfacial engineering, because the same effect can be produced by a catalytic powder. A stronger criterion is a change in response when chemical composition is held constant, and only the spatial organization of contact is altered [62,63,69,81,82,83].

Such a control was implemented for GO/Cu-MOF. At a heating rate of 5 K min^−1^, spray-assembled AP@GO/Cu-MOF gave an HTD of 331.27 °C compared with 335.63 °C for the mechanical mixture, corresponding to an interface-organization contribution of 4.36 °C [63]. Heat release decreased slightly, while E_a_ for the high-temperature stage decreased from 163.52 to 139.10 kJ mol^−1^, and that for the low-temperature stage increased. A more homogeneous contact therefore does not simply ‘accelerate decomposition’; rather, it redistributes kinetic limitations among sequential stages. The frequently cited 105.49 °C shift in this study refers to pre-calcined GO/Cu-MOF-300 and does not characterize the intact MOF.

In the solvent–nonsolvent ZIF-8/AP nanocomposite, 10 wt.% ZIF-8 lowered HTD by 81.48 °C and nearly doubled heat release [62]. Here, ZIF-8 acts both as an energetic component and as a precursor to ZnO, which forms directly within the reaction zone. Similarly, 1 wt.% MIL-88B(Fe) shifted the main AP exothermic peak by approximately 71 °C and increased ΔH from 733 to 1218 J g^−1^, although E_a_ determined by Kissinger and Kissinger–Akahira–Sunose (KAS) analyses decreased by only a few percent [69]. A large temperature shift accompanied by only a moderate change in E_a_ suggests that the MOF affects not only the formal kinetic barrier but also the distribution of reaction sites, heat transfer, and the location at which the catalytic oxide phase forms. The same dual functionality is evident for the solvent-free nitrogen-rich [Co(HTATT)]_n_ framework, which was characterized as an energetic material in terms of thermal decomposition, calculated detonation performance, and mechanical sensitivity and was subsequently tested as a promoter of AP decomposition [84]. A recent solvent-free three-dimensional Cu(I)-EMOF provides a similar example, combining high thermal stability and low mechanical sensitivity with measurable promotion of AP decomposition [85].

Under simple physical mixing, the same sequence is retained, but the role of interface geometry is more difficult to isolate. At 5 wt.%, ZIF-67 lowered AP HTD from approximately 425 to 335 °C and increased heat release from 784.8 to 2282 J g^−1^ [81]. AP first oxidizes the energetic framework, after which the resulting Co-containing products accelerate conversion of the remaining oxidizer. Cu_2_(bdc)_2_(bpy) and Cu_3_(BTC)_2_ also show strong catalytic responses at low loadings, but different concentrations optimize different parameters [82,83]. Recent Cu-BTC data make this loading dependence explicit. Across 1–5 wt.% Cu-BTC, the strongest thermal response occurred at 1 wt.%: at 10 K min^−1^, the AP high-temperature decomposition peak decreased by 119.5 °C, the apparent activation energy decreased by 94.4 kJ mol^−1^, and total heat release increased by 396.1 J g^−1^. Direct combustion tests additionally showed a non-monotonic loading dependence, with excessive Cu-BTC reducing performance because of mass-transfer blockage [86]. Morphology-controlled Zn-MOF_x_ provides a complementary mesoscale comparison: modification of the growth environment generated distinct MOF morphologies, and the most active variant lowered the AP high-temperature decomposition peak by 99.2 °C while increasing heat release by 469.3% [87]. For a 2-methylimidazole-modified Zn-MOF, the high-temperature AP decomposition peak shifted from 322.5 to 296.5 °C and heat release increased to 905.6 J g^−1^, whereas the ignition delay remained close to that of the parent framework—21 versus 18 ms [34]. For Cu_3_(BTC)_2_, additional evidence of practical relevance is its good compatibility with AP according to the vacuum stability test (VST) at 100 °C for 40 h. Thus, in powder-contact systems, the chemical activity of the MOF is better established than the advantage of a specific interfacial topology.

A different interfacial regime is obtained when the external energetic phase is a molecular explosive rather than an inorganic oxidizer. Cu-MOF shells have been grown around RDX, HMX, and CL-20 crystals to form core–shell energetic composites while retaining the nitramine as the energetic core [88]. MOF coating shifted the thermal decomposition behavior, substantially increased the apparent activation energy, and reduced both impact and friction sensitivity, while calculated detonation performance was largely retained. In this case, EF acts primarily through modification of the local mechanical and thermal environment of the energetic crystal, including rigid shielding and suppression of localized mechanical activation, rather than through the AP-type catalytic pathway discussed above.

A distinct regime arises when the MOF is no longer a minor additive but becomes the principal fuel component. In the stoichiometric [Cu(atrz)_3_(NO_3_)_2_]_n_/AP composition containing 36.3 wt.% MOF, the ignition temperature was 242 °C, the heat of reaction was 3.84 kJ g^−1^ by DSC and 5.41 kJ g^−1^ by bomb calorimetry, and the maximum pressure (P_max_) reached 6.9 MPa [89]. Here, the interface becomes a coupled fuel–oxidizer reaction zone in which catalytic and energetic functions cannot be separated. Improved ESD and friction sensitivity were accompanied by poorer impact sensitivity, underscoring the multidimensional nature of safety-related performance in such a composition.

Overall, MOF interfaces with oxidizers and energetic crystals span several distinct regimes, from catalytic powder contact and deliberately organized oxidizer interfaces to core–shell protection of molecular explosives and complete fuel-oxidizer reaction zones. The relevant EF variable therefore depends on the system: contact organization and sequential reaction kinetics for oxidizers, local stress and heat-transfer conditions for nitramine crystals, and coupled chemistry when the MOF itself becomes a major energetic component. In all cases, the intact framework defines the initial interfacial state and may subsequently transform in situ into catalytically or energetically active products.

### 3.4. MOFs in Polymer and Composite Matrices: Active-Phase Accessibility and Behavior of the Final Formulation

In a formulated pyrotechnic or rocket-propellant composition, the polymer matrix is not merely an inert binder but actively influences reaction-zone organization and transport processes. It determines the distances between reactants, oxidizer access to the MOF, heat and mass transfer, additive migration, and moisture uptake. Testing of the formed material is therefore particularly important, because the properties of the free MOF may change markedly after incorporation into a polymer or propellant grain [64,65,70,90,91].

This behavior is clearly illustrated by n-Al/poly(vinylidene fluoride) (PVDF)/EMOF. The mechanical mixture burned at approximately 0.5 m s^−1^, whereas electrospun EF@E(DHBT) reached approximately 2.8 m s^−1^ and released 3464 J g^−1^ compared with 1616 J g^−1^ [64]. A fiber control without MOF indicates that the acceleration occurs in two stages: the one-dimensional morphology shortens transport distances, while the surface-bound MOF further enhances the reaction. PVDF simultaneously etches Al_2_O_3_, while EMOF decomposition generates gas and suppresses sintering [64]. These observations are consistent with a positive feedback process in which exposure of fresh surface accelerates transport, and faster transport promotes further surface exposure.

The importance of spatial integration is also evident in boron-containing ternary systems. Compared with physically blended B@PVDF/EMOF, in situ growth of the energetic MOF on B@PVDF produced a B@PVDF@EMOF architecture with shorter ignition delay and improved combustion efficiency. The optimized B@PVDF@EMOF-72 sample reached a maximum heat release of 21,387 J g^−1^ [78]. This comparison reinforces that the polymer phase can participate in the engineered interface rather than acting only as an inert binder.

A paraffin matrix represents the opposite limit. Free Co(AIm)_2_ and Co(VIm)_2_ ignite with white fuming nitric acid (WFNA) within milliseconds, but homogeneous incorporation of 20 wt.% Co(VIm)_2_ into paraffin does not provide reliable ignition [65]. Localized placement of approximately 10 mg of MOF on the surface or in a small cavity restores short delays—12.9/6.3 ms for Co(VIm)_2_ and 2.3/1.8 ms for Co(AIm)_2_. The nominal concentration is therefore less important than the actually exposed fraction of active surface: the matrix can either deliver the functionality to the oxidizer or completely shield it. Moreover, the appearance of first light does not necessarily guarantee sustained flame transfer across the entire paraffin sample.

Hydroxyl-terminated polybutadiene (HTPB) affects not only accessibility but also the chemical sequence. In the MOF-235/Fe-MOF–AP system, pristine MOF-235 appears more active and has a much larger Brunauer–Emmett–Teller surface area; however, after HTPB is introduced, the second-stage temperatures for MOF-235/AP/HTPB and Fe-MOF/AP/HTPB converge to 345.9 and 342.7 °C, respectively [70]. In formed samples, both materials increased the burning rate by approximately 20%. This suggests that once the binder is present, the rate-limiting step shifts from the porosity of the pristine MOF toward the chemistry of the combined MOF/HTPB/AP reaction zone: HTPB pyrolysis products participate in transport between AP and the forming Fe_2_O_3_@C phase [70].

Formulation-level evidence from Fe-MOF/AP/HTPB and CoMg-MOF systems further shows that intact frameworks can transform in situ into dispersed oxide- or carbon-containing products while retaining measurable effects on heat release and burning behavior [92,93]. Importantly, this formulation-level role is not restricted to AP-based propellants. A mixed energetic-MOF system composed of Bi-MOF and CuFe-MOF in a 2:1 ratio produced the strongest cooperative thermal response toward both RDX and HMX, with reported synergy indices of 3.35 and 3.24, respectively [94]. When transferred to an RDX-containing composite modified double-base propellant (RDX-CMDB) together with a small amount of carbon black, the system increased burning rate by up to 80.6% and reduced the pressure index by up to 59.6%. In HMX-containing nitrate-ester-plasticized polyether (HMX-NEPE) propellants, the pressure index decreased from 0.65 to 0.38 for the non-aluminized formulation and from 0.50 to 0.40 for the aluminized formulation. These results demonstrate that MOF-derived kinetic effects identified at the nitramine level can remain relevant after incorporation into substantially more complex propellant matrices.

A more mechanistically resolved example is provided by RDX-CMDB formulations containing heterobimetallic PbBa-MOF or NiBa-MOF. PbBa-MOF produced a pressure index of approximately 0.18 and extended the low-pressure-exponent combustion region to approximately 10–20 MPa [95]. In situ solid-phase FTIR and gas-phase MS–FTIR associated the improved combustion response with accelerated C–N bond cleavage in RDX and C–C bond cleavage in the NG/NC matrix, followed by enhanced thermal feedback between the condensed combustion surface and the gas-phase reaction zone. This example extends MOF analysis beyond isolated thermal decomposition to the combustion-wave behavior of an actual modified double-base propellant.

The matrix effect is even more evident for [Cu(atrz)_3_(NO_3_)_2_]_n_. In a model AP/catalyst system, CuO lowered HTD more strongly than the Cu-MOF; however, in a complete composite solid propellant, the MOF produced a lower final decomposition temperature and a stronger burning response [90]. For the MOF-containing formulation, the pressure exponent decreased to n = 0.424, mechanical sensitivity decreased, and the theoretical specific impulse (I_sp_) and characteristic velocity (C*) changed only slightly at MOF contents up to 5 wt.%. Thus, in this formulation, the energetically active framework showed an advantage over the conventional nonenergetic catalyst at the full-composition level even though the simplified AP test did not predict this behavior.

The role of the matrix is not always to accelerate the reaction. A K-containing α-cyclodextrin MOF in nitrocellulose (NC) forms an extended network of electrostatic and hydrogen-bonding interactions, decreases additive mobility, and reduces moisture uptake to 1.335% compared with 1.526–1.563% for K_2_SO_4_/KNO_3_ controls [91]. At the same time, the flame area at 3 MPa decreases to 78.25 mm^2^ compared with 102.41 mm^2^ for NC/KNO_3_. In this case, the beneficial interfacial effect lies not in maximum acceleration but in more stable component distribution, lower moisture uptake, and suppression of an undesirable flash.

A more formulation-oriented extension of this concept was recently demonstrated by in situ self-assembly of CD-K-MOF on a surface-denitrated nitrocellulose-based propellant. The resulting bifunctional surface architecture combined a gradient denitration layer with a porous MOF barrier, producing progressive combustion with a dynamic vivacity increment (ΔL) of 0.0791 MPa^−1^ s^−1^ while reducing the muzzle-flash area by 54%. The potassium-rich MOF additionally suppressed CO and NO_x_ emissions, and the overall thermal response was described as a retardant-then-catalytic process [96].

Thus, the matrix acts as a kinetic boundary condition that determines whether the intrinsic activity of the MOF is realized in the final formulation. Spatial structuring can greatly accelerate combustion, encapsulation can suppress hypergolicity, HTPB can change the relative effectiveness of catalysts, and strong intermolecular interactions with NC can stabilize a component and reduce flame intensity. For the matrix-interface EF, the key parameter is not maximum MOF loading but its accessibility and localization relative to the process that is intended to be enhanced or suppressed.

## 4. Comparative Analysis of Internal-Framework and Interfacial Functionalization

### 4.1. From Functionalization Site to the Rate-Limiting Stage

Comparison of IF and EF shows that these strategies primarily govern different levels of the same reaction chain. IF determines the intrinsic chemical capabilities of the MOF: how readily charge can be transferred, which redox states are accessible, how much energetic content is carried by the ligand or guest, and how stable the framework remains before initiation. EF determines where and how rapidly this functionality is expressed in the formulation through contact with the oxidizer, metallic fuel, conductive phase, or polymer matrix.

The boundary between IF and EF is therefore defined not by the component with which the material is tested, but by the variable changed in the comparative experiment. If the ligand, node, or internal guest is modified while AP serves only as a probe of the response, the system remains an IF case. If MOF chemistry is retained while the mode of contact with Al, B, AP, or the matrix is changed, EF becomes the determining level. This classification helps avoid conflating intrinsic reactivity with interfacial delivery.

The strongest examples show that the two strategies can operate sequentially. An acetylene-containing ligand establishes millisecond hypergolicity within the framework [11], an Fe–Co node provides more favorable redox chemistry [24], and an ionic liquid within Ni-MOF opens an electrically driven initiation pathway [27]. At the EF level, however, contact geometry determines whether this potential is realized: n-Al@MIL-53(Al) reproduces rapid ignition only when the shell is formed [67], Cu-ATRZ@Al/APTES outperforms the physical mixture [72], and electrospun Al/PVDF/EMOF greatly accelerates combustion relative to randomly mixed components [64].

The IF/EF distinction identifies the predominant site of modification but does not imply that the resulting mechanisms are independent. Coupling between the two levels may generate emergent behavior, understood here as a structural state or reaction pathway that cannot be predicted from their separate responses without accounting for their interaction. In such systems, the interface can feed back into the framework state, extending the role of EF beyond the delivery of pre-existing functionality.

The systems discussed above illustrate this interdependence. In the GO-containing Zn–5-aminotetrazolate system, interfacial coordination stabilized a metastable polymorph, whereas physical mixing with GO did not provide comparable stabilization [60]. In Fe-based MOF/AP/HTPB systems, comparisons of binary and ternary mixtures revealed a redistribution of decomposition stages, and the authors proposed a feedback mechanism involving MOF transformation products and binder decomposition [70]. These observations support structural and kinetic coupling, respectively, but do not independently establish emergence from separately controlled IF and EF modifications.

Assessment of IF/EF combinations should therefore distinguish performance enhancement, non-additive interaction, and mechanistic emergence. Where the modifications can be varied independently, comparisons should include a common reference, IF-only, EF-only, and combined IF/EF systems under matched conditions and with consistent normalization. A combined response that deviates beyond experimental uncertainty from a clearly defined independent-effects prediction would support an interaction for the selected metric. Establishing mechanistic emergence would additionally require evidence of the distinct structural state or reaction pathway responsible. Improved performance relative to either individual modification alone is insufficient to establish either non-additive interaction or mechanistic emergence. Accordingly, sequential IF/EF design remains a useful starting point, while coupled architectures require joint evaluation because interfacial interactions may alter the framework response and the rate-limiting stage.

### 4.2. Data Comparability, Standardized Evaluation, and Strength of Evidence

Quantitative comparison of functionalization strategies is limited primarily by nonuniform test conditions. For AP, studies differ in MOF mass fraction, heating rate, atmosphere, mixing procedure, and the characteristic temperature reported—onset, low-temperature decomposition, or HTD peak. Activation energies additionally depend on the kinetic model and conversion range [97]. Consequently, cross-study ranking based on an absolute temperature shift or a single E_a_ value is rarely physically justified; within-series comparisons performed under the same protocol are more reliable.

To visualize the available quantitative evidence without implying direct equivalence among studies, Figure 5 compares the reported AP high-temperature decomposition (HTD) shift as a function of MOF/additive content for IF and EF systems.

Across the studies summarized in Figure 5, IF systems exhibited AP HTD reductions of approximately 6–116 °C at reported additive loadings of 2–10 wt.%, whereas EF systems showed reductions of approximately 73–111 °C at 5 wt.%. Even at the same nominal loading of 5 wt.%, IF results ranged from approximately 6 to 116 °C, indicating that additive content alone does not explain the observed variation [24,43,99]. For EF, varying the GO fraction within GO@MOF-5 yielded shifts of approximately 73–88 °C [68], while ZIF-67@CNTOH achieved a shift of approximately 111 °C [100]. Thus, the reported IF and EF ranges overlap substantially, providing no clear evidence of a universal advantage of either strategy under the heterogeneous experimental conditions represented.

To improve cross-study comparability, a minimum standardized evaluation protocol should be adopted for MOF-based energetic systems. Before energetic testing, the phase identity and purity of the MOF, particle size and morphology, activation or guest state, functional-phase loading, and method of spatial integration should be documented [101]. For thermal analysis, mandatory reporting should include sample mass, additive loading, heating rate, atmosphere and gas-flow rate, crucible type, the definition of the reported thermal event (Tonset, LTD, or HTD peak), and the kinetic-analysis method and conversion range [97]. Ignition and combustion studies should specify formulation composition, specimen geometry and mass, pressure and atmosphere, ignition source and input energy, recording or imaging conditions, and the definitions used for ignition delay and burning rate. Sensitivity measurements should identify the test method or standard, sample conditioning, number of replicates, and threshold criterion. Where direct comparisons are intended, these conditions should be matched as closely as possible. Controls should also correspond to the mechanistic question: IF studies should include the parent or structurally comparable framework, whereas EF studies should include a physical mixture or a compositionally matched system without the deliberately engineered interface; formulation-level studies should additionally include the corresponding catalyst-free or reference formulation [24,63,67]. Where complete harmonization is not feasible, explicit reporting of these parameters is essential for separating material-dependent effects from protocol-dependent variability.

To provide a quantitative side-by-side comparison while preserving these methodological distinctions, Table 3 summarizes representative IF and EF effects on decomposition temperature, heat release, burning rate, and sensitivity. Numerical values are referenced to the corresponding within-study controls, and multiple studies are included where suitable data are available. The comparison is therefore intended to identify metric-specific trends and trade-offs rather than to establish a universal superiority of either functionalization strategy.

Taken together, the quantitative evidence supports a functional distinction between the two levels of modification. IF changes the intrinsic chemical response through linker, node, or guest engineering, whereas EF more directly modifies the efficiency with which heat, mass, and charge are transferred within the composite system. However, the magnitude and even the direction of the resulting change remain strongly dependent on material composition, formulation, and test method.

The same applies to energetic quantities. Heat of combustion, heat of decomposition, enthalpy of formation, and calculated Q_det_ describe different processes; calculated detonation velocity D and detonation pressure P from EXPLO5 should not be placed on the same evidentiary scale as experimental ignition delay, burning rate, or sensitivity [29,36,38,49]. Accordingly, improvement in a calculated energetic metric should not automatically be described as improved overall performance.

Finally, the state of the MOF before and during reaction must be distinguished. In this review, a system is classified as IF or EF when an intact framework is introduced into the formulation; formation of oxide, metallic, or carbonaceous products during heating is treated as part of its reaction mechanism. Pre-calcined or pyrolyzed materials are instead classified as MOF-derived systems and are excluded. If an independent oxide phase is already present before testing, as in the mechanochemical Co–Fe system, the observed response belongs to a multiphase material, rather than to pure framework functionalization.

### 4.3. Performance Trade-Offs and Practical Design Constraints

Negative results and performance trade-offs are equally important for defining the practical limits of MOF functionalization. Internal-framework modification can improve energetic output while simultaneously reducing safety margins. For example, replacing nitrate with dinitramide in Cu–ATRZ increased the calculated heat of detonation from 4562 to 7176 kJ kg^−1^, but decreased the impact-, friction-, and electrostatic-discharge thresholds from 16 to 9 J, 112 to 73 N, and 24.75 to 1.9 J, respectively [26]. Thus, increasing energetic density through guest or counterion engineering can shift the system toward a less favorable sensitivity regime. A different trade-off is observed for interfacial functionalization of nano-Al: formation of an in situ Al-MOF surface shell shortened the ignition delay from 20 to 4 ms and strongly increased the pressure-rise rate, but reduced the combustion heat from 22.32 to 17.06 kJ g^−1^ because part of the active-metal fraction was replaced by the interfacial layer [67]. These examples show that neither IF nor EF should be evaluated using a single maximized response.

Failure cases further show that the effectiveness of a functionalization strategy is conditional on architecture, loading, and formulation context. In the nB/EMOF system, the MOF fraction that maximized one response did not maximize the others: the strongest DSC response in air occurred near 10 wt.% EMOF, maximum calorimetric heat release in O_2_ at 25 wt.%, and the fastest flame propagation at 30 wt.% [75]. Thus, there is no universally optimal loading independent of the target metric. Spatial organization can also determine whether functionalization is effective at all. Physical mixtures of nano-Al with the corresponding ligand or MOF did not reproduce the ignition response of the directly formed Al-MOF shell [67], while physical mixing of GO with MOF(ATA-a) failed to provide the structural stabilization obtained when GO was incorporated during MOF crystallization [60]. Conversely, in a simplified AP/catalyst test, CuO lowered the HTD more strongly than Cu-MOF, yet in the complete AP/Al/HTPB formulation the MOF gave the more favorable final decomposition and burning response [90]. These cases define an important practical boundary: results obtained for isolated thermal decomposition, physical mixtures, or a single loading cannot be assumed to predict performance in the final energetic formulation.

Environmental impact represents an additional practical design constraint. Reticular chemistry provides opportunities to reduce reliance on toxic heavy-metal centers through the use of lower-toxicity metal nodes or heavy-metal-free coordination architectures [9]. A representative lower-toxicity example is the Zn(II)-based zeolite-like energetic framework IFMC-1, which combines high thermal and air stability with low impact, friction, and electrostatic-discharge sensitivity [35]. A more direct heavy-metal-free strategy is demonstrated by the potassium-based frameworks [K(CHT)]_n_ and [K(AOT)]_n_, which contain no transition-metal center and exhibit decomposition temperatures of 314 and 310 °C, respectively, together with IS > 40 J and FS > 360 N [36]. These examples indicate that greener MOF design can be pursued without necessarily sacrificing thermal stability or safety. However, the designation of a “green energetic material” should extend beyond replacement of Pb-, Ag-, or other toxic metal centers: metal-node toxicity, ligand and oxidizer composition, synthesis route, decomposition products, and the environmental persistence of residues should be considered together. Lower-toxicity and heavy-metal-free coordination chemistries therefore represent an important design direction, but their environmental advantages should ultimately be evaluated across both synthesis and end-use stages.

### 4.4. Storage Stability, Aging Behavior, and Environmental Adaptability

Environmental adaptability is particularly important for MOF-containing energetic formulations because exposure to water, humidity, corrosive species, and component migration may alter the framework itself as well as its interface with the surrounding energetic matrix [102]. In nitrocellulose, incorporation of a K-containing cyclodextrin MOF reduced moisture uptake to 1.335%, compared with 1.526–1.563% for conventional K_2_SO_4_/KNO_3_ controls, while also suppressing additive migration and crystalline precipitation [91]. This indicates that MOF incorporation can improve environmental stability through intermolecular immobilization rather than simply through catalytic activity. A complementary fuel-interface strategy was demonstrated for Al-TCPP-coated flake-Al. The MOF coating provided pronounced resistance to chloride-induced corrosion during prolonged exposure to 3.5 wt.% NaCl solution while largely preserving the combustion calorific value of the metallic fuel [74]. These examples show that environmental adaptability may arise either from stabilization of a polymeric energetic matrix or from protection of a reactive metallic-fuel surface.

A representative example is the graphene-oxide-encapsulated metastable Zn–5-aminotetrazolate framework, GO⊂MOF(ATA-a) [60]. Pristine MOF(ATA-a) undergoes complete polymorphic transformation to the more stable MOF(ATA-b) during storage at room temperature and undergoes the same transition after 7.5 h at 120 °C. In contrast, GO⊂MOF(ATA-a) remained structurally stable in air for three months and retained its framework after heating at 120 °C for 24 h, whereas physical mixing with GO did not provide comparable stabilization. The authors attributed this enhanced stability to extensive coordination interactions between oxygen-containing groups on GO and Zn sites within the MOF. This example demonstrates that interfacial functionalization can suppress thermally and temporally driven structural evolution of an energetic MOF before initiation [60]. More generally, however, a high decomposition temperature alone should not be regarded as evidence of thermal durability, because conventional thermogravimetric measurements may not capture structural transformations that occur without substantial mass loss [103].

Systematic thermal-cycling studies involving repeated heating–cooling sequences remain scarce within the intact-MOF energetic literature surveyed here. Consequently, the retention of crystallinity, interfacial integrity, mechanical sensitivity, ignition behavior, and combustion performance after repeated temperature excursions remains largely unresolved. This distinction is important because thermal stability measured during a single heating experiment is not equivalent to resistance to repeated thermal stress. Future studies should therefore combine cyclic temperature exposure with post-cycling structural characterization and energetic-performance measurements to determine whether both the MOF and the engineered interface remain functional.

Long-term shelf-life data are likewise considerably less developed than thermal-decomposition and short-duration combustion measurements. Most MOF-based energetic studies reviewed here characterize freshly prepared materials, whereas relatively few follow the same formulation after extended storage under controlled temperature and humidity. High initial crystallinity, a high decomposition temperature, or low mechanical sensitivity therefore cannot by themselves establish long-term service stability. Shelf-life assessment should additionally monitor structural retention, moisture uptake, additive migration, corrosion or passivation of metallic fuels, changes in mechanical sensitivity, and retention of ignition and combustion performance over time. Accelerated-aging protocols should ideally combine controlled temperature and relative humidity with post-aging PXRD, DSC/TG, chemical-compatibility testing, sensitivity measurements, and formulation-level combustion tests. The scarcity of such longitudinal datasets currently represents an important qualification gap for MOF-functionalized energetic materials.

### 4.5. Principles for Selecting a Functionalization Strategy

Selection between internal-framework and interfacial functionalization should begin not with the MOF type, but with identification of the stage that limits performance of the energetic system. If the bottleneck is the intrinsic reactivity of the framework—electronic structure, redox chemistry, energetic loading, or the state of an internal guest—IF is favored. If the reaction is instead limited by oxidizer access, disruption of a passivating layer, heat transfer, agglomeration, or spatial accessibility of the active phase within the binder, EF is more rational. A complex combined architecture is justified only when these limitations operate simultaneously. This approach changes the optimization criterion itself: functionalization should remove a specific kinetic or transport limitation rather than merely introducing another active component.

Operationally, the stage that limits performance should be treated as the dominant performance-limiting process under a defined set of conditions rather than as a universal elementary rate-determining step. It can be identified through matched perturbation experiments in which one level of the system is varied while the others are kept as constant as practically possible. If changes in linker or node chemistry at comparable loading and morphology alter apparent kinetics and the distribution of gaseous intermediates, an intrinsic chemical limitation is implicated; the Fe/Co/Fe–Co MOF series, in which thermal, combustion, and gas-phase FT-IR responses changed coherently, provides an example [24]. Conversely, if comparable components exhibit different responses when the spatial organization of contact or the surrounding matrix is changed, an interfacial or transport limitation is implicated. This is illustrated by spray-assembled AP@GO/Cu-MOF versus the corresponding mechanical mixture [63], by n-Al@MIL-53(Al), for which physical mixtures did not reproduce the response of the directly formed MOF shell [67], and by Al/PVDF/EMOF and Fe-MOF/AP/HTPB systems, where morphology or binder incorporation altered the controlling regime [64,70]. Accordingly, DSC/TG peak temperatures and apparent E_a_ should be interpreted together with evolved-gas or in situ phase analysis [24,93] and formulation-level observables such as ignition delay, burning rate, and pressure development [24,64,67]; kinetic parameters should be evaluated using consistent analysis procedures [97]. Because the controlling process can shift with conversion and formulation, the limiting stage should be assigned for the relevant operating regime rather than treated as a fixed property of the MOF. Based on these considerations, the selection of a functionalization strategy can be organized as a conditional pathway that begins with the target performance response and then identifies the dominant process limiting that response. Intrinsic chemical limitations favor IF, interfacial or transport limitations favor EF, whereas simultaneous limitations may require a combined IF/EF design. This conceptual selection pathway is summarized in Figure 6.

The conceptual selection pathway is summarized in Figure 6, whereas Table 4 provides a more detailed comparison of the corresponding functionalization approaches, dominant mechanisms, expected performance gains, limitations, and applicable energetic scenarios.

### 4.6. Priorities for Future Research

The next step for the field is to move beyond comparison of final thermoanalytical metrics toward direct, time-resolved identification of the reaction sequence. In many systems considered here, the initially intact MOF acts only at an early stage and subsequently transforms in situ into oxide, metallic, or carbon-containing phases [33,62,92,93]. Mechanistic conclusions should therefore be weighted according to the directness of the experimental evidence. Temperature-resolved in situ or operando X-ray diffraction can identify loss of the parent framework and the appearance of crystalline intermediate or product phases [104], whereas simultaneous TG–MS or TG–FTIR can correlate individual mass-loss stages with the evolution of specific gaseous products [105]. Within the energetic-MOF literature reviewed here, TG–IR analysis of Fe–Co-MOF/AP resolved changes in NH_3_ adsorption and N_2_O/NO_x_ evolution [24], while in situ solid-phase FTIR combined with gas-phase MS–FTIR in MOF-containing RDX-CMDB formulations linked combustion behavior to accelerated C–N cleavage in RDX and C–C cleavage in the NG/NC matrix [95]. High-speed imaging provides complementary temporal evidence for initiation and combustion-front propagation [52,64]. Combining these techniques with thermography and pressure–time measurements would connect framework transformation and gas evolution to ignition, flame propagation, and macroscopic energy release. Because temperature-resolved in situ XRD remains underrepresented in the energetic-MOF literature surveyed here, its wider use, together with simultaneous TG–MS/TG–FTIR, represents an important priority. Mechanistic assignments based only on DSC/TG peak shifts should therefore be regarded as provisional unless supported by phase- or product-resolved evidence.

Equally important is the transition from model MOF/AP pairs to formed energetic formulations. Studies of electrospun Al/PVDF/EMOF fibers and Cu-MOF-containing AP/HTPB propellants show that an effect observed in a simple mixture can be amplified, weakened, or even reverse the relative ranking of materials after a binder and fuel are introduced [64,90]. AP decomposition temperature should therefore be treated as an intermediate indicator rather than as an endpoint performance metric. For practical evaluation, at least one thermal parameter should be linked to a measured formulation-level response: ignition delay, burning rate, pressure exponent, P_max_ or pressure-rise rate (dP/dt), completeness of metallic-fuel oxidation, and the nature of the residue.

Metallic fuels beyond Al and B represent a particularly clear opportunity for extending the IF/EF framework. In the literature surveyed here, direct peer-reviewed studies in which intact MOFs are deliberately used to functionalize metallic Mg or Zr particles remain substantially less developed than corresponding Al- and B-based systems. This distinction is important: Mg- or Zr-containing MOFs, in which these elements form part of the framework node, should not be interpreted as MOF-functionalized Mg or Zr fuels. Future studies should therefore determine whether reactive or protective MOF interfaces can regulate passivation-layer stability, oxidizer access, ignition threshold, aging behavior, and combustion propagation in Mg- and Zr-containing pyrotechnic formulations.

Engineering translation should be assessed separately from laboratory-scale energetic performance. For MOF technologies more generally, scale-up is governed by precursor and linker cost, solvent and reagent consumption, synthesis temperature and duration, product yield, purification and activation requirements, throughput, batch-to-batch reproducibility, and the ability to preserve particle size and morphology during larger-scale production [106,107]. Energetic applications impose additional constraints, including sensitivity during synthesis and handling, compatibility with conventional mixing, casting, or pressing operations, storage stability, and reproducibility of coating or interface geometry at increased batch size. Within the present literature, systems already evaluated in complete propellant or energetic formulations—such as MOF/AP/HTPB, AP/Al/HTPB, RDX-CMDB, and MOF-modified nitrocellulose systems [70,90,91,94,95,96]—provide stronger evidence of application readiness than powder-level thermal tests alone. However, this does not establish a general technological advantage of EF over IF: simple low-loading framework modifications may be easier to manufacture than multistep core–shell or guest-loaded architectures. The most promising candidates for near-term engineering translation are therefore those that combine reproducible synthesis or assembly, limited processing complexity, low or moderate functional-phase loading, formulation-level performance gains, and acceptable sensitivity and storage behavior. Formal TRL assignment is not yet justified for most energetic-MOF systems because pilot-scale production, manufacturing cost, long-term reliability, and process-safety data remain scarce.

Future progress will therefore require simultaneous advances in three directions: standardized experimental design and reporting, direct time-resolved evidence for the operative mechanism, and explicit evaluation of scalability, cost, process safety, and formulation-level reliability. Moving from the rate-limiting stage toward the required architecture, rather than from an available MOF toward an arbitrarily selected performance metric, provides a more transferable basis for both fundamental design and engineering translation.

## 5. Conclusions

This review has examined functionalization of intact metal–organic frameworks for pyrotechnic and related energetic compositions through two complementary structural levels: internal-framework functionalization and external/interfacial functionalization. This distinction provides a mechanistic basis for connecting a specific structural modification with the stage of the energetic transformation that it controls. IF determines the intrinsic chemical preparedness of the framework through linker electronics and energetic content, metal-node redox chemistry, pore and guest environments, and coupled modifications of these structural elements. In contrast, EF governs how this intrinsic functionality is expressed within a multicomponent formulation by controlling contact with metallic or boron-containing fuels, oxidizers, conductive carbon phases, and polymer matrices.

The reviewed studies demonstrate that neither approach can be considered intrinsically superior. Linker and node engineering can accelerate initiation or decomposition, alter energetic output, and modify sensitivity, while pore and guest functionalization can introduce additional optical, electrical, or chemical initiation pathways. At the interfacial level, deliberate spatial organization can shorten transport distances, disrupt passivating Al_2_O_3_ or B_2_O_3_ layers, improve heat and charge transfer, suppress agglomeration, and position reactive species directly within the reaction zone. However, improvement in one response does not necessarily translate into better overall energetic performance. Lower decomposition temperatures, reduced apparent activation energies, higher calculated energetic characteristics, faster ignition, and increased burning rates represent different stages of the reaction sequence and may be accompanied by changes in sensitivity, stability, compatibility, or active-fuel fraction.

A central conclusion is therefore that MOF functionalization should be selected according to the rate-limiting process of the target energetic formulation rather than according to the MOF structure alone. When intrinsic electronic structure, redox chemistry, energetic loading, or guest behavior limits performance, IF provides a more direct design route. When the limiting processes involve oxidizer access, passivation-layer disruption, heat or mass transfer, particle agglomeration, or accessibility of the active phase within a binder, EF becomes more relevant. In systems where both limitations operate, the most rational strategy is sequential: first establish the required chemical reactivity within the framework and then spatially position this functionality at the interface where it can most effectively influence the reaction.

Further progress will require moving beyond isolated thermoanalytical indicators toward direct formulation-level validation. Thermal measurements should increasingly be linked with ignition delay, burning rate, pressure development, metallic-fuel oxidation, sensitivity, residue composition, and long-term compatibility. Time-resolved in situ characterization, gas-product analysis, high-speed imaging, thermography, and pressure measurements will be particularly important for distinguishing the role of the initial MOF from that of oxide, metallic, or carbonaceous phases generated during reaction. Equally important are matched control materials, standardized reporting of composition and testing conditions, and systematic evaluation of storage stability and safety.

## Figures and Tables

**Figure 1:**
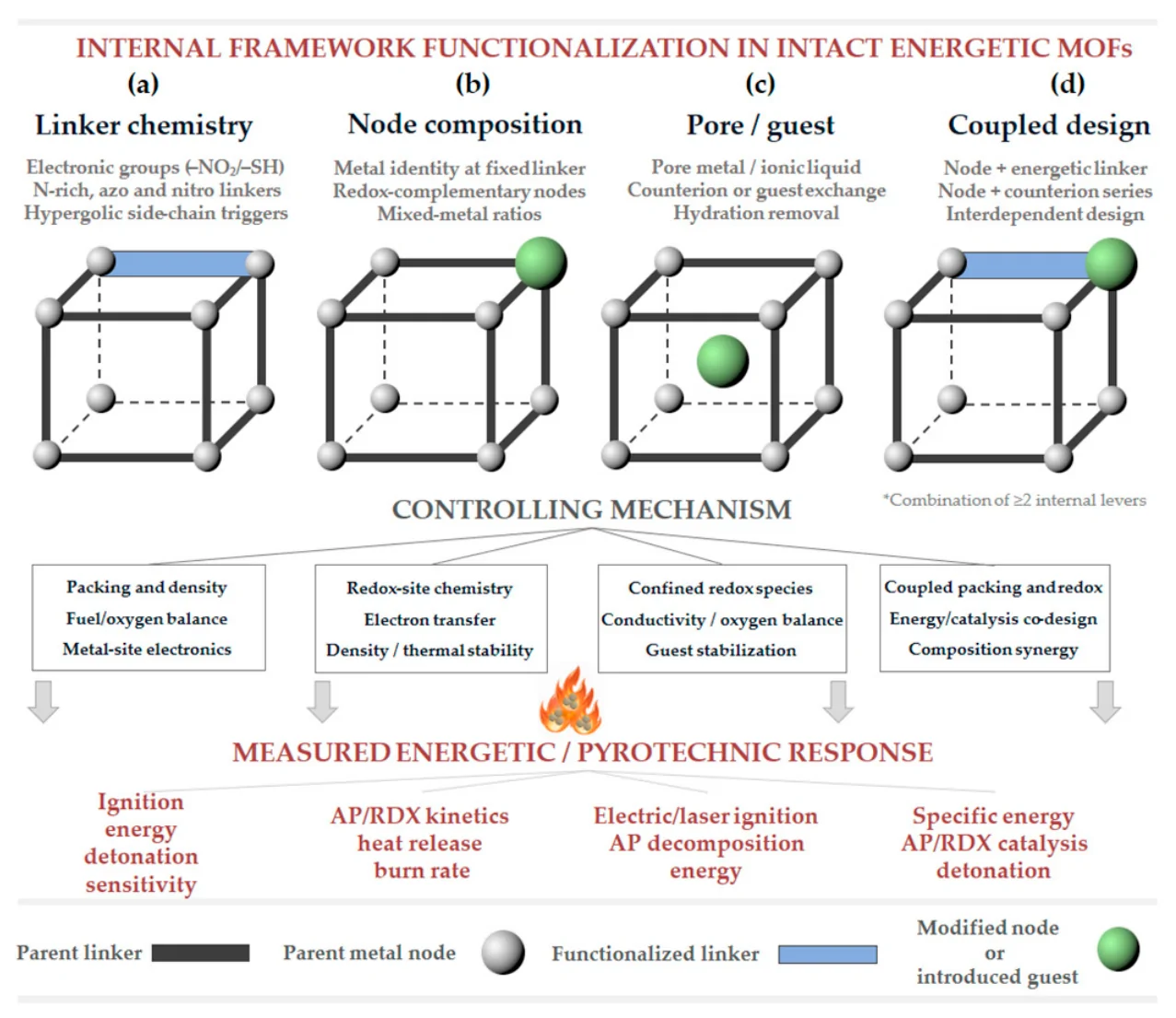
Internal-framework functionalization strategies in intact energetic metal–organic frameworks (MOFs): linker chemistry, node composition, pore/guest engineering, and coupled internal design, together with their governing mechanisms and experimentally evaluated energetic/pyrotechnic responses. The asterisk (*) indicates the combination of two or more internal functionalization strategies. Created by the authors based on the concepts and experimental findings reported in Refs. [11,23,24,25,26,27,28,29].

**Figure 2:**
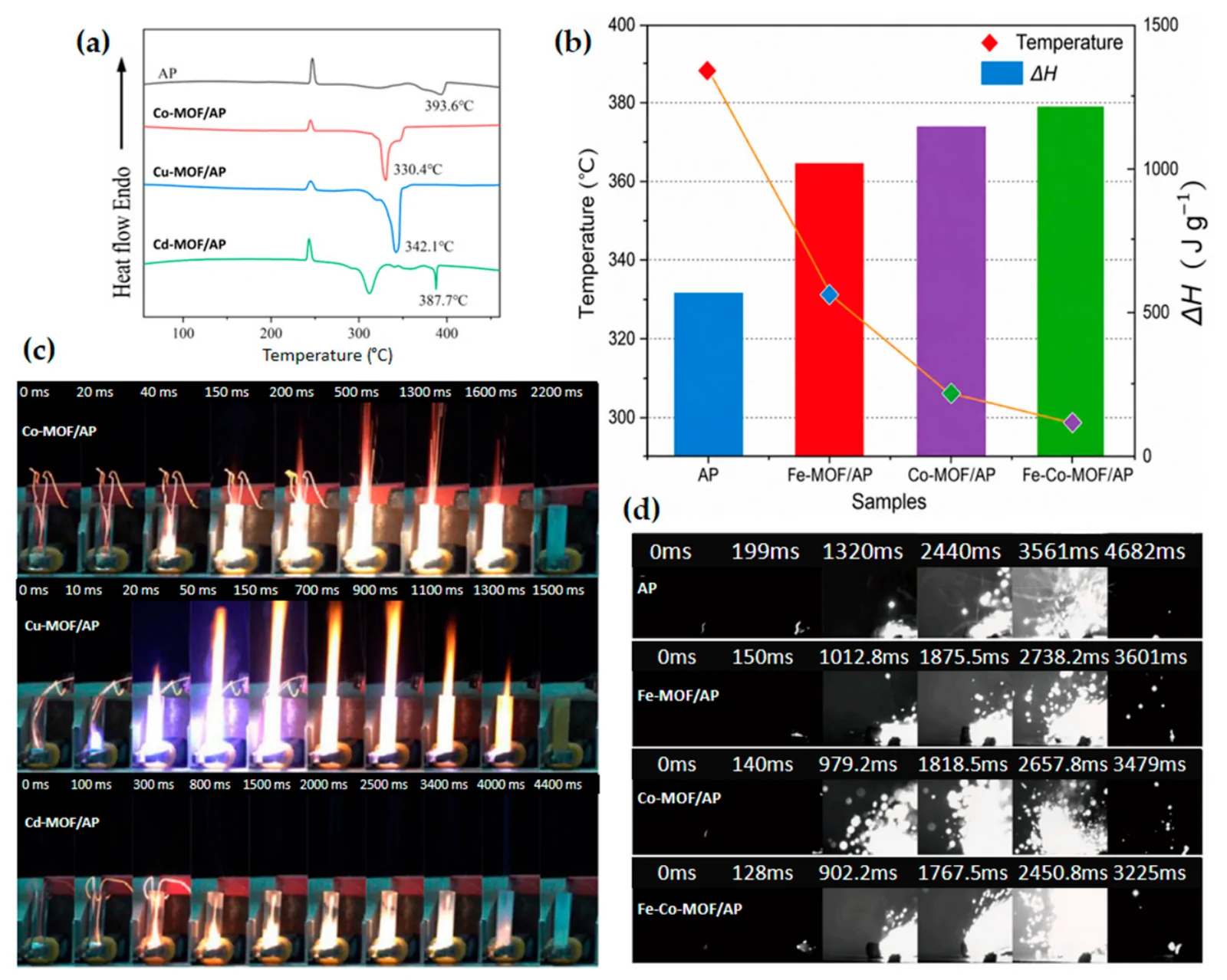
Effects of metal-node functionalization in intact MOFs on ammonium perchlorate decomposition and combustion: (**a**) DSC curves of AP containing Co-, Cu-, and Cd-based MOFs; (**b**) decomposition temperature and heat release of AP containing Fe-, Co-, and Fe–Co-MOFs; (**c**) time-resolved combustion images of Co-, Cu-, and Cd-MOF/AP mixtures; and (**d**) combustion sequences of AP and Fe-, Co-, and Fe–Co-MOF/AP mixtures. In (**a**), the upward arrow indicates the endothermic direction of heat flow. In (**b**), the blue, red, purple, and green bars represent the heat release (ΔH) of AP, Fe-MOF/AP, Co-MOF/AP, and Fe–Co-MOF/AP, respectively. The diamond markers connected by the orange line indicate the corresponding decomposition peak temperatures. Panels (**a**,**c**) adapted from [43]; panels (**b**,**d**) adapted from [24].

**Figure 3:**
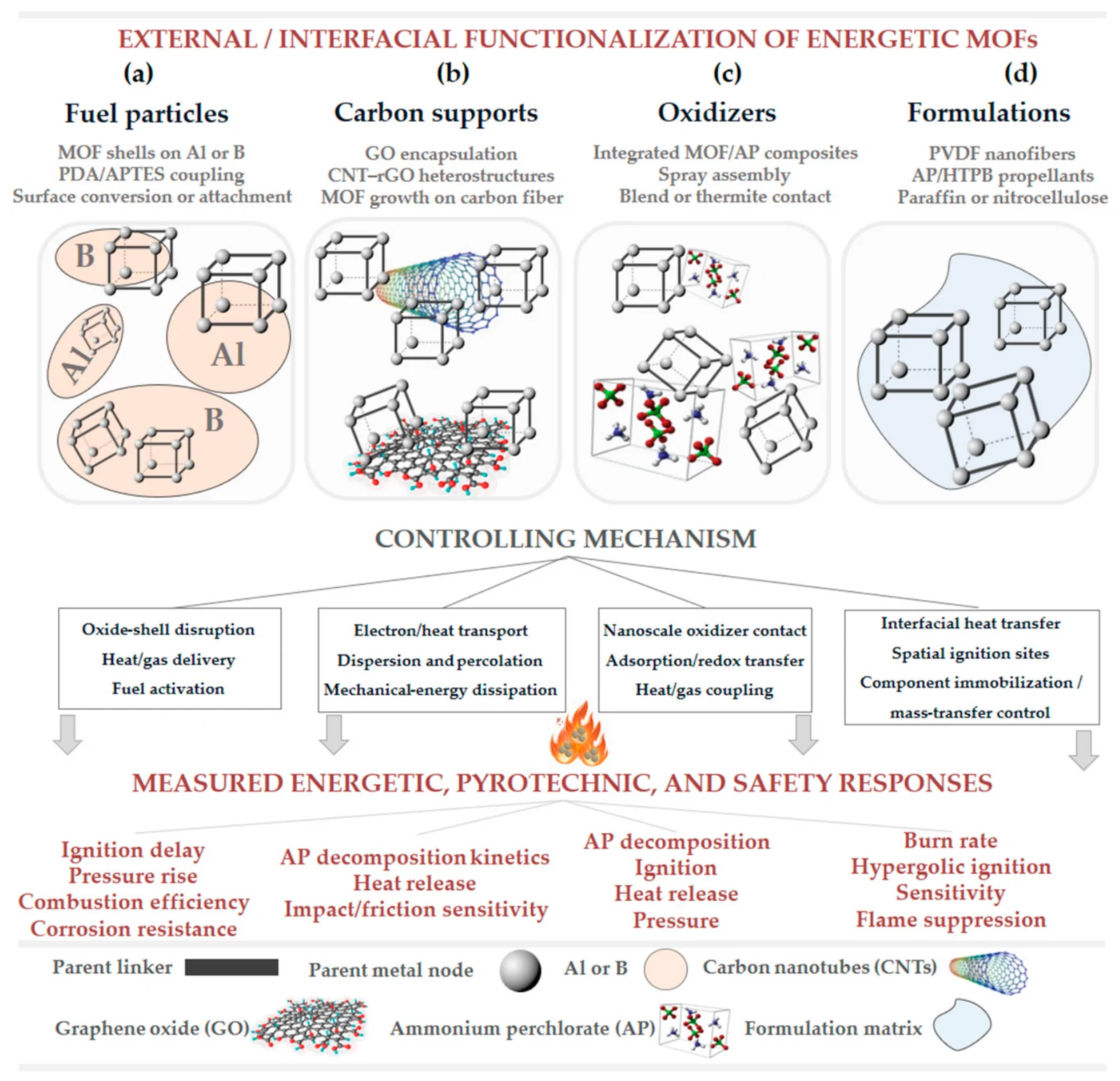
External and interfacial functionalization strategies of energetic metal–organic frameworks through fuel-particle, carbon-support, oxidizer, and formulation interfaces, together with the corresponding mechanisms governing ignition, decomposition, combustion, pressure generation, and safety-related responses. Created by the authors based on the concepts and experimental findings reported in Refs. [58,59,60,61,62,63,64,65].

**Figure 4:**
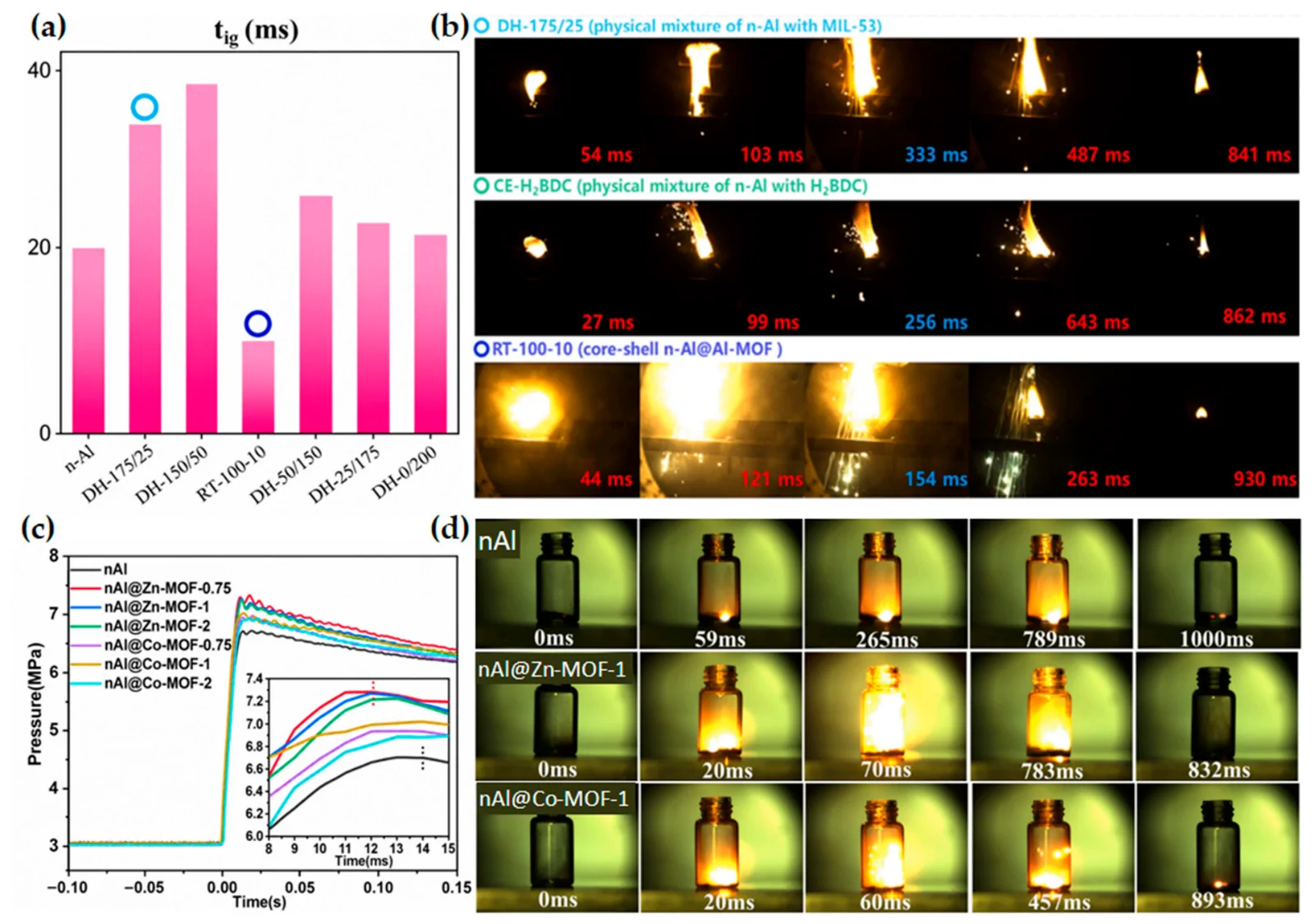
Effects of external MOF functionalization of aluminum particles on ignition and combustion: (**a**) ignition-delay time of pristine n-Al, physical mixtures, and core–shell n-Al@Al-MOF; (**b**) combustion sequences of physical mixtures and core–shell n-Al@Al-MOF; (**c**) pressure–time profiles of pristine n-Al and Zn- or Co-MOF-coated aluminum particles; and (**d**) combustion sequences of n-Al, n-Al@Zn-MOF, and n-Al@Co-MOF. In (**a**), the light-blue and dark-blue circles highlight the physical-mixture and core–shell samples, respectively, selected for combustion comparison in (**b**). In (**c**), the differently colored dotted lines distinguish the pressure–time profiles of pristine n-Al and the corresponding Zn- and Co-MOF-coated samples, as indicated in the legend. Panels (**a**,**b**) reproduced from [67]; panels (**c**,**d**) reproduced from [76].

**Figure 5:**
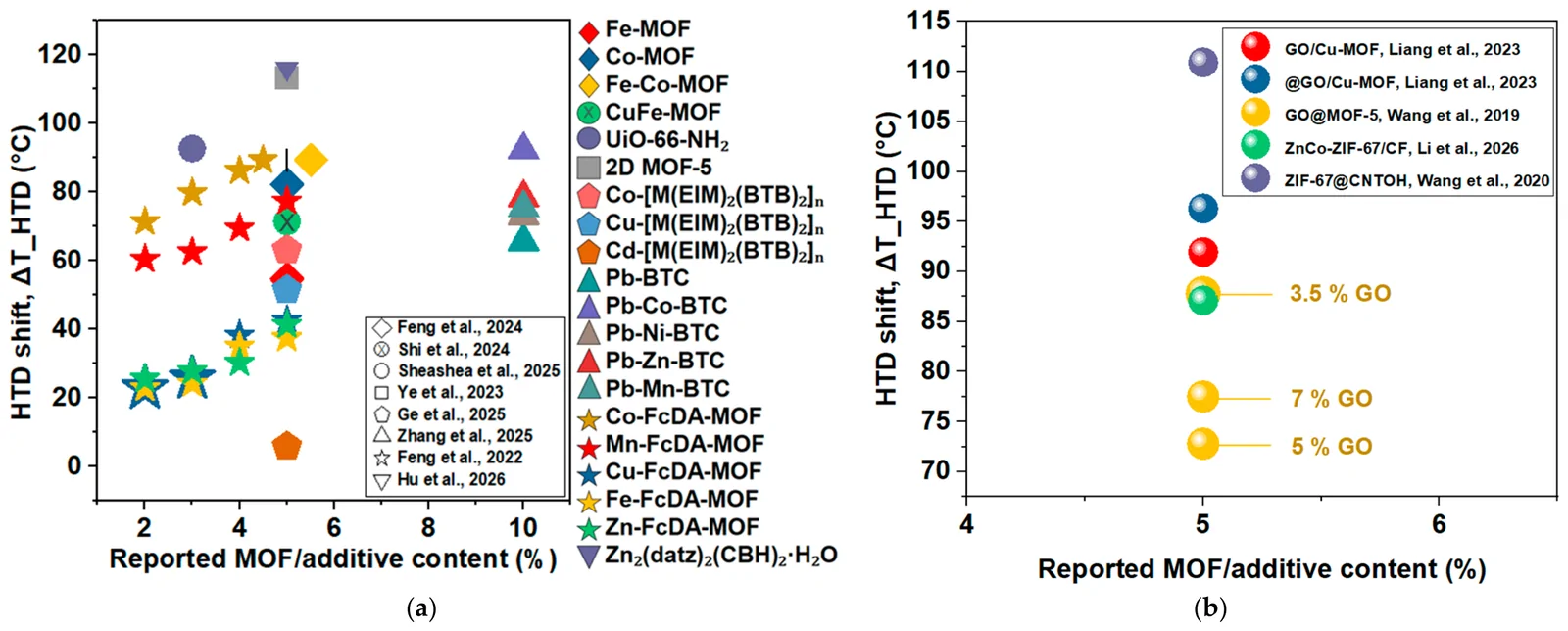
Reported shifts in the high-temperature decomposition (HTD) peak of ammonium perchlorate as a function of MOF/additive loading for (**a**) internal-framework (IF) [24,31,33,34,43,50,98,99] and (**b**) external/interfacial (EF) functionalization systems [63,68,79,100]. Colors identify individual MOF compositions, whereas marker shapes indicate the corresponding references. In panel (**a**), the black line connects two markers with identical reported loading (5 wt.%) and HTD shifts (89.4 °C); one marker was horizontally displaced to prevent overlap. Differences in experimental conditions limit direct quantitative comparisons between studies.

**Figure 6:**
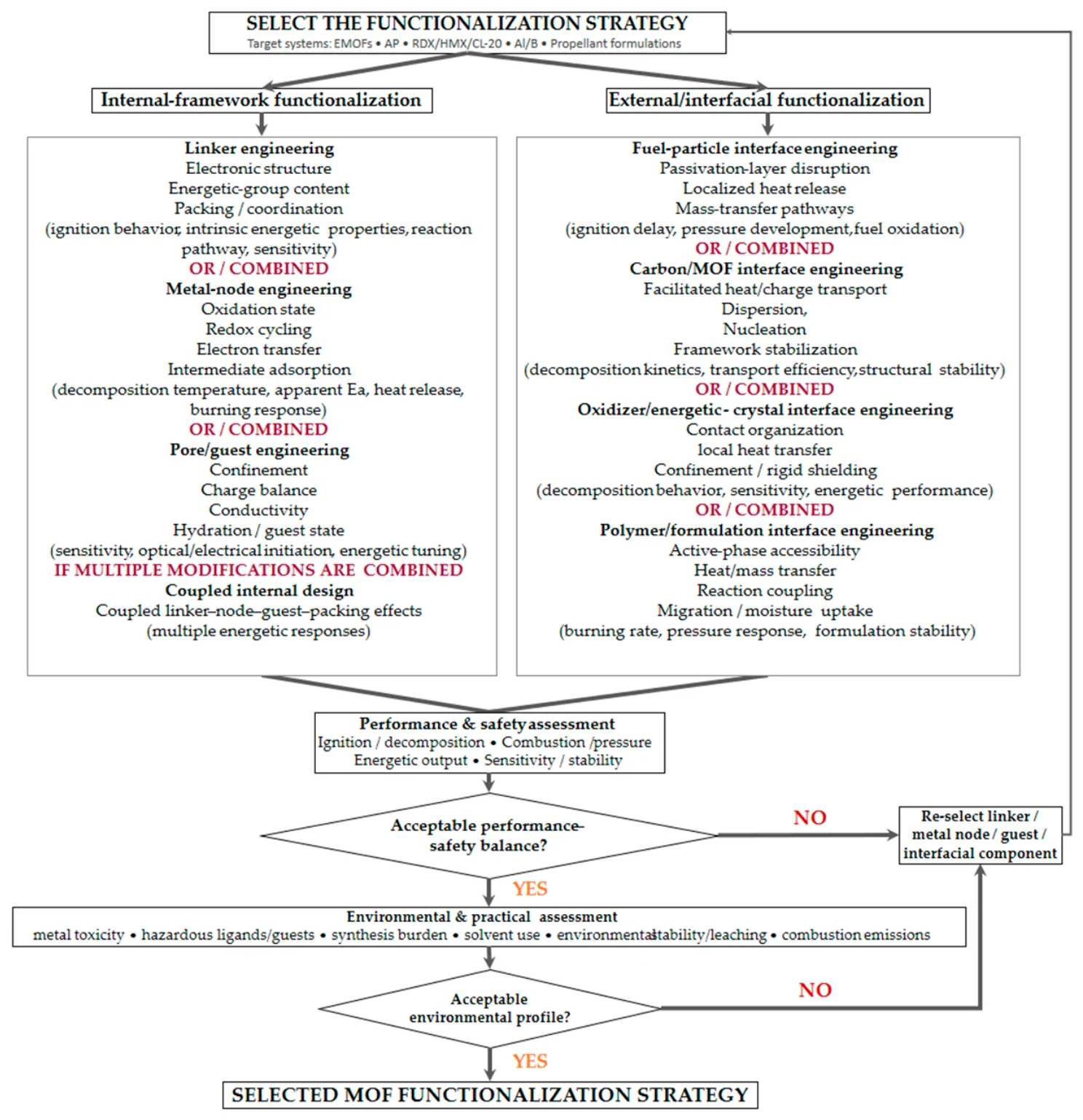
Conceptual decision flowchart for selecting internal-framework or external/interfacial functionalization in MOF-based energetic systems according to the dominant performance-limiting process. When intrinsic and interfacial limitations coexist, both branches should be evaluated sequentially. The selected strategy should be validated in the relevant formulation and refined according to performance–safety trade-offs.

**Table 1:** Representative Internal-Framework Functionalization Strategies and Energetic Responses of Intact Metal–Organic Frameworks.

MOF/Functionalization	Energetic System	Representative Comparative Result	Ref.
Linker-centered functionalization			
Zn/Co/Cd ZIFs; vinyl → acetylene linker	WFNA hypergolic ignition	Ignition delay: 11–35 → 2–5 ms; free ligand ≈ 34 ms	[11]
Cu(I)-MOFs; NO_2_/SH linker electronics	[BMIM]N(CN)_2_/H_2_O_2_ bipropellant	Ignition delay: 120 → 50 → 18.4 ms	[23]
Node-centered functionalization			
Fe-, Co-, and Fe–Co-MOF-74; mixed-metal nodes	AP-based propellant	Burning rate increase: +43.8% for Fe–Co-MOF	[24]
Co/Cu/Pb dicyanamide MOFs; metal identity at fixed linker	MOF/AP system	AP T_dec_: 379.2 → 318.3/306.5/340.3 °C	[25]
Pore/guest functionalization			
Cu-ATRZ; dinitramide counterion exchange	Energetic MOF	Q_det_ (calc.): 4562 → 7176 kJ kg^−1^; IS: 16 → 9 J	[26]
IL@Ni-MOF; ionic-liquid pore loading	Electrically controlled solid propellant	Ignition delay at 0.5 wt.%: 1.158 → 0.722 s; optimum 0.336 s	[27]
Cu/Mn-bis(tetrazolyl)methane; NH_3_OH^+^ incorporation	High-energy-density material	Combustion energy: 11.447/11.510 MJ kg^−1^	[30]
Coupled internal functionalization			
Mn/Co framework with energetic DCA linker	AP/RDX system	RDX E_a_: 153.0 → 86.3 kJ mol^−1^	[28]
CuFe-MOF; coupled heteronuclear-node/energetic-linker design	RDX/HMX/CL-20/AP	At 5 wt.%: apparent E_a_ decreased by 38.5/38.3/63.9/27.0 kJ mol^−1^, respectively	[31]

**Table 2:** Representative External and Interfacial Functionalization Strategies and Energetic Responses of MOF-Based Systems.

MOF/Interfacial Functionalization	Energetic System	Representative Comparative Result	Test Conditions	Ref.
Fuel-particle interfaces				
B@PDA@R-MOF(MA); MOF shell on nano-B	Boron ignition/combustion	Combustion efficiency: 60.7 → 80.4%	20 wt.% MOF; oxygen-bomb calorimetry	[59]
nB@MOF; Zn/Co/Mo node-tunable MOF interfaces	Boron oxidation/combustion	Combustion heat > 1.2 × pristine nB	Self-assembled nB@MOF; TG–DSC, laser ignition, constant-volume combustion	[66]
n-Al@MIL-53(Al); native oxide → MOF surface conversion	Nano-Al ignition/combustion	Ignition delay: 20 → 4 ms	RT-100-120; CO_2_ laser, 15 W, 1000 ms; ~10 mg; air	[67]
Carbon-supported heterointerfaces				
Cu-MOF/CNT-rGO; conductive carbon/MOF heterointerface	AP decomposition	AP HTD: 409.7 → 313.8 °C	5 wt.% additive; DSC, 10 °C min^−1^; N_2_, 30 mL min^−1^	[61]
GO@MOF-5; graphene oxide/MOF heterointerface	AP decomposition	AP HTD: 409.7 → 321.9 °C	5 wt.% GO@MOF-5; DSC, 10 °C min^−1^; N_2_, 30 mL min^−1^	[68]
MOF/oxidizer interfaces				
ZIF-8/AP; integrated MOF/AP nanocomposite	AP decomposition	HTD decrease: 81.48 °C	ZIF-8:AP = 1:9; DSC, 10 °C min^−1^; N_2_, 50 mL min^−1^	[62]
MIL-88B(Fe)/AP; integrated MOF/oxidizer system	AP decomposition	Main decomposition peak: −71 °C	1 wt.% MOF; DSC, 10 °C min^−1^; N_2_, 50 mL min^−1^	[69]
Formulation and polymer interfaces				
n-Al/PVDF/EMOF; electrospun multi-interface nanofiber	Energetic composite	Burning rate: 2.8 m s^−1^, ≈5× that of the mechanical mixture	EF@EMOF(DHBT); high-speed-camera combustion test	[64]
Fe-MOF/AP/HTPB; MOF–oxidizer–binder interface	Composite solid propellant	Burning rate increase: +20%	2 wt.% MOF; 5 × 5 × 30 mm strips; Ni–Cr ignition; air	[70]

**Table 3:** Metric-specific comparison of internal-framework (IF) and external/interfacial (EF) functionalization effects in MOF-based energetic systems.

Metric	Strategy	Representative System/Functionalization	Quantitative Result	Ref.
Decomposition temperature	IF	Cu–ATRZ framework; internal NO_3_^−^ → N(NO_2_)_2_^−^ counterion exchange	Onset decomposition temperature: 223 → 221 °C	[26]
	IF	Pb–BTC with Co/Ni/Zn/Mn species introduced into the framework pores	Pure AP: 430.8 °C; parent Pb–BTC/AP: 365.1 °C; Pb–Co-BTC/AP: 338.4 °C; Pb–Ni-BTC/AP: 357.6 °C; Pb–Zn-BTC/AP: 352.4 °C; Pb–Mn-BTC/AP: 355.4 °C	[50]
	EF	Cu-MOF@CNT-rGO/AP heterointerface	AP HTD: 409.7 → 313.8 °C	[61]
Heat release	IF	Fe-, Co-, and Fe–Co-MOF node engineering in AP	DSC heat release: pure AP 576 J g^−1^ → Fe-MOF/AP 1013.84 J g^−1^, Co-MOF/AP 1151 J g^−1^, Fe–Co-MOF/AP 1213 J g^−1^	[24]
	IF	Co-, Cu-, and Pb-based M[N(CN)_2_]_2_ frameworks with fixed dicyanamide linker	Experimental combustion heat: 2184.3, 1916.0, and 996.7 kJ mol^−1^ for Co-, Cu-, and Pb-based frameworks, respectively	[25]
	EF	Cu-MOF@CNT-rGO/AP	DSC heat release: 576.0 → 1333.8 J g^−1^	[61]
	EF	MIL-88B(Fe)/AP	DSC heat release: 733 → 1218 J g^−1^ (+66%)	[69]
	EF	n-Al with in situ Al-MOF surface shell, RT-100-10	Combustion heat: 22.32 → 17.06 kJ g^−1^ (−23.6%)	[67]
Burning rate	IF	Co-, Fe-, and Fe–Co-MOF node engineering in AP-based composite propellant	Burning-rate increase relative to catalyst-free propellant: +29.5%, +32.1%, and +43.8%, respectively	[24]
	IF	Co-, Cu-, and Cd-based borane MOF fuels substituted for Al in composite propellants	MOF-containing propellants: 8.1–13.4 mm s^−1^ vs. Al-containing control: 7.1–9.4 mm s^−1^ at 3–9 MPa	[43]
	EF	Electrospun n-Al/PVDF/EMOF nanofibers	Burning rate: ~0.5 m s^−1^ (mechanical mixture) → ~2.8 m s^−1^ (electrospun composite); >5-fold increase.	[64]
	EF	Fe-MOF or MOF-235 incorporated into AP/HTPB propellant	Burning rate increased by approximately 20% relative to catalyst-free propellant	[70]
Sensitivity	IF	Cu–ATRZ; internal dinitramide counterion exchange	Impact sensitivity: 16 → 9 J; friction sensitivity: 112 → 73 N; ESD threshold: 24.75 → 1.9 J	[26]
	IF	[K(CHT)]_n_ and [K(AOT)]_n_ energetic MOFs obtained by internal ligand design	Both MOFs: IS > 40 J; FS > 360 N	[36]
	EF	GO⊂MOF(ATA-a) obtained by GO encapsulation during MOF crystallization	Pristine MOF, physical GO/MOF mixture, and GO⊂MOF composite: IS > 40 J; FS > 360 N; ESD > 24.75 J	[60]
	EF	[Cu(atrz)_3_(NO_3_)_2_]_n_ incorporated into AP/Al/HTPB composite propellant	Friction sensitivity: 40 → 32%; impact sensitivity H_50_: 39.4 → 63.5 cm	[90]

Note: Numerical values represent within-study comparisons and should not be interpreted as direct IF–EF comparisons. DSC heat release and combustion heat are reported separately because of methodological differences. Higher sensitivity thresholds indicate lower sensitivity; “>” denotes an experimental lower bound.

**Table 4:** Comparative decision matrix for IF and EF strategies in MOF-based energetic systems.

Functionalization Approach	Modification Site	Dominant Mechanism	Key Performance Gains	Main Limitations/Trade-Offs	Applicable Scenarios	Representative Refs.
IF—linker engineering	Organic linker/substituent	Electronic redistribution, energetic-group incorporation, packing regulation	Tunable ignition, intrinsic energy, reaction-pathway modulation	Increased reactivity may increase sensitivity; ligand synthesis and packing effects complicate prediction	Intrinsic initiation chemistry or energetic content is limiting	[11,23,32,41]
IF—metal-node engineering	Metal identity, oxidation state, or mixed-metal node	Redox cycling, electron transfer, adsorption/conversion of intermediates	Lower apparent activation energy and/or decomposition temperature, increased heat release, improved burning response	Different metrics may favor different metals; toxicity and phase evolution must be considered	Redox-controlled oxidizer decomposition	[24,25,44]
IF—pore/guest engineering	Pore environment, counterion, guest, hydration state	Confinement, charge balance, conductivity, guest-triggered initiation	Desensitization, controlled optical/electrical initiation, energetic tuning	Guest retention, hydration sensitivity, energy–sensitivity trade-offs	Confinement-, conductivity-, or trigger-limited systems	[26,27,48,51,53]
IF—coupled internal design	Multiple framework variables	Coupled linker/node/packing/guest effects	Simultaneous tuning of several energetic responses	Difficult mechanistic deconvolution; demanding matched controls and reproducibility	Multiple intrinsic limitations operate simultaneously	[28,31,56]
EF—fuel-particle interface	MOF/Al or MOF/B surface	Passivation-layer disruption, localized heat release, shortened mass-transfer/diffusion paths	Shorter ignition delay, faster pressure development, enhanced fuel oxidation	Active-fuel dilution, coating uniformity, possible decrease in gravimetric combustion heat	Passivation- or transport-limited metallic fuels	[58,59,66,67,78]
EF—carbon/MOF interface	MOF/carbon boundary	Charge and heat transport, dispersion, nucleation control, framework stabilization	Faster decomposition, improved transport, structural stabilization	Carbon contribution may be difficult to separate; dispersion/agglomeration effects	Heat- or charge-transfer-limited systems	[60,61,68,79,80]
EF—oxidizer/energetic-crystal interface	MOF/oxidizer or MOF/nitramine boundary	Reaction-zone organization, local heat transfer, confinement or rigid shielding	Decomposition control, sensitivity mitigation in core–shell energetic crystals, preserved energetic performance	Strong loading/morphology dependence; model thermal tests may not predict final formulation behavior	Contact-limited oxidizer decomposition or mechanically sensitive energetic crystals	[62,63,69,88]
EF—polymer/formulation interface	MOF–binder–energetic matrix	Accessibility control, matrix-mediated heat/mass transfer and reaction coupling	Burning-rate and pressure control, reduced migration/moisture uptake, formulation-level performance	Binder may shield or redirect MOF activity; effects are strongly formulation-specific	Final propellants and formulated pyrotechnic systems	[64,65,70,78,90,91,94,95,96]

Note: Mechanisms and performance outcomes are system-dependent. Interfacial contributions may not be independently verified, and overlapping categories do not represent independent evidence.

## Data Availability

No new data were created or analyzed in this study. Data sharing is not applicable to this article.

## Abbreviations

The following abbreviations are used in this manuscript:

AP	Ammonium perchlorate
APTES	(3-Aminopropyl)triethoxysilane
ATRZ	4,4′-Azo-1,2,4-triazole
CNT	Carbon nanotube
CMDB	Composite modified double-base
DCA	Dicyanamide
DSC	Differential scanning calorimetry
E_a_	Apparent activation energy
EF	External/interfacial functionalization
EMOF	Energetic metal–organic framework
ESD	Electrostatic-discharge sensitivity
FS	Friction sensitivity
GO	Graphene oxide
HTD	High-temperature decomposition
HTPB	Hydroxyl-terminated polybutadiene
IF	Internal-framework functionalization
IL	Ionic liquid
IS	Impact sensitivity
MOF	Metal–organic framework
PDA	Polydopamine
P_max_	Maximum pressure
PVDF	Poly(vinylidene fluoride)
Qdet	Heat of detonation
RDX	Cyclotrimethylenetrinitramine
rGO	Reduced graphene oxide
WFNA	White fuming nitric acid
